# Engineered Nanocatalyst‐Enabled Cheolesterol Depletion for Enhanced Tumor Piezocatalytic Therapy

**DOI:** 10.1002/advs.202500967

**Published:** 2025-02-18

**Authors:** Mengdan Li, Yajun Zhou, Xiaoyang Chen, Weiwei Chen, Yifei Yang, Cheng Qian, Lei Yang, Yaohan Zhang, Zheng Zhang, Wencheng Wu, Yifei Yin

**Affiliations:** ^1^ Department of Medical Ultrasound Affiliated Hospital of Nantong University, Medical School of NanTong University Nantong Jiangsu 226001 P. R. China; ^2^ Department of Ultrasound The Fourth Affiliated Hospital Nanjing Medical University Nanjing Jiangsu 210029 P. R. China; ^3^ Department of Medical Ultrasound Affiliated Hospital of Jiangsu University Zhenjiang Jiangsu 212000 P. R. China; ^4^ Central Laboratory and Department of Medical Ultrasound Sichuan Academy of Medical Sciences Sichuan Provincial People's Hospital University of Electronic Science and Technology of China Chengdu Sichuan 610072 P. R. China

**Keywords:** cholesterol depletion, piezocatalytic tumor therapy, reactive oxygen species, tumor antimetastatic

## Abstract

The inadequate generation of reactive oxygen species (ROS) and metastasis of malignant tumors are critical factors that limit the efficacy of conventional sonodynamic therapy in cancer treatment. Herein, an engineered piezocatalyst: cholesterol oxidase (CHO)‐loaded Pt‐ZnO nanoparticles (Pt‐ZnO/CHO) that can explosively generate large amounts of ROS and block the metastasis of tumor, is developed for improving piezocatalytic tumor therapy. In this process, Pt‐ZnO can substantially generate ROS via initiating ultrasound (US)‐triggered piezocatalytic reactions. In situ‐grown Pt nanoparticles not only optimize piezocatalytic activities but also facilitate oxygen (O_2_) production, thereby synergistically boosting ROS generation. Moreover, O_2_ produced by Pt‐ZnO can accelerate the depletion of excess cholesterol in tumor cells under CHO catalysis to disrupt the integrity of lipid rafts and inhibit the formation of lamellipodia, significantly suppressing the proliferation and metastasis of tumor cells. This strategy by promoting ROS generation and blocking the metastatic pathway of cancer cells offers a new idea for enhanced efficacy‐oriented cancer therapeutic strategies.

## Graphical abstract

Under ultrasound (US) irradiation, cholesterol oxidase (CHO)‐loaded Pt‐ZnO nanoparticles (Pt‐ZnO/CHO) initiates a piezo‐catalytic cascade reaction that produces oxygen (O_2_) and reactive oxygen species (ROS), leading to the consumption of cholesterol, disruption of lipid raft integrity, and interference with lamellipodia formation. This process inhibits epithelial‐mesenchymal transition (EMT), suppressing tumor migration and invasion, and achieving an effective piezo‐catalytic anticancer effect.

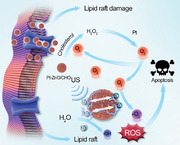

## 1 Introduction

Recently, sonodynamic therapy (SDT) has emerged as a promising modality for anticancer treatment.^[^ ^1^, ^2^, ^3^ ^]^ This approach involves the activation of sonosensitizers by US, resulting in the release of energy and the subsequent generation of reactive oxygen species (ROS), which are instrumental in inducing cytotoxic effects on tumor cells.^[^ ^4^, ^5^ ^]^ However, the efficiency of ROS generation using conventional sonosensitizers is strongly dependent on the oxygen (O_2_) content within tumor tissues. The hypoxic tumor microenvironment (TME) significantly impedes ROS production, thereby diminishing therapeutic efficacy.^[^ ^6^ ^]^ Furthermore, the ongoing depletion of O_2_ during treatment exacerbates the development of drug resistance in tumors.^[^ ^7^ ^]^ Piezoelectric catalysts, classified as non‐centrosymmetric crystals, possess the ability to release electrons and holes in response to external mechanical strain, such as ultrasound (US) irradiation.^[^ ^8^, ^9^ ^]^ This process facilitates the catalytic conversion of substrate H_2_O or O_2_ to produce ROS, including hydroxyl radicals (·OH) and superoxide anions (·O_2_^−^).^[^ ^10^ ^]^ In contrast to conventional acoustic sensitizers, which rely on the consumption of O_2_ for ROS generation, piezoelectric materials exhibit a reduced dependence on O_2_, enabling ROS production through electron transfer pathways involving reactions with H_2_O or O_2_.^[^ ^9^ ^]^ Meanwhile, the rational engineering of piezoelectric catalysts can endow them with enzyme‐like activity to catalyze the generation of O_2_ from Hydrogen peroxide (H_2_O_2_) overexpressed in the tumor, thereby enhancing the efficiency of ROS generation in hypoxic tumors.^[^ ^11^ ^]^ Therefore, the in situ activation of intratumoral piezoelectric catalytic reactions represents an effective strategy to overcome TME limitations, facilitating substantial ROS generation and thereby improving the catalytic therapeutic efficacy against cancer.

In addition, the post‐treatment metastasis of tumors is the leading cause of cancer‐related death worldwide, yet the treatment for tumor metastasis is still unsatisfactory.^[^ ^12^ ^]^ While the disruption of the TME induced by ROS can delay tumor progression, it fails to address the issues of tumor migration and invasion that are prompted by elevated cell motility.^[^ ^13^, ^14^ ^]^ It has been demonstrated that the movement and migration of tumor cells are intrinsically linked to the formation of lamellipodia and the integrity of lipid rafts.^[^ ^15^, ^16^ ^]^ Lamellipodia are broad, flattened protrusions located at the leading edge of cells, which play a critical role in sensing the cellular environment and facilitating cell movement. Their formation necessitates the assembly of the actin cytoskeleton and the dynamics of membrane movement.^[^ ^17^ ^]^ Lipid rafts, characterized as tightly packed microdomains rich in cholesterol and sphingolipids within the plasma membrane, are capable of regulating tumor cell migration by mediating the internalization and recycling of cell surface proteins.^[^ ^18^ ^]^ Furthermore, lipid rafts are a prerequisite for the formation of choriocapillaris, which would be inhibited when the integrity of lipid rafts is disrupted.^[^ ^19^ ^]^ Cholesterol has been implicated in the facilitation of a critical biological process during invasion and metastasis, specifically epithelial‐mesenchymal transition (EMT).^[^ ^20^ ^]^ EMT is characterized by the transformation of epithelial‐derived tumor cells, which exhibit limited invasive and metastatic potential, into mesenchymal‐like tumor cells with enhanced invasive and metastatic capabilities.^[^ ^21^ ^]^ Transforming growth factor (TGF)‐β is widely recognized for its role in regulating various cellular processes, including cell proliferation, differentiation, and migration.^[^ ^22^ ^]^ Therefore, disrupting lipid rafts of tumor cells and inhibiting EMT by depleting cholesterol may represent a viable strategy for inhibiting tumor cell metastasis.

**Scheme 1:**
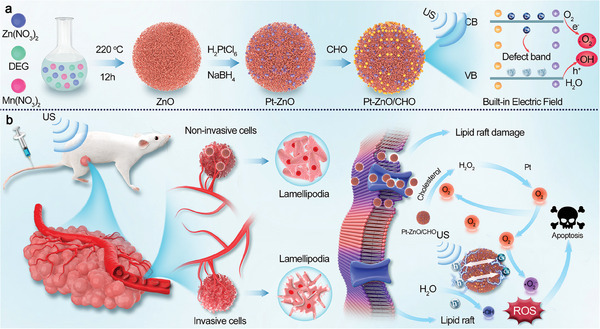
Schematic illustration of Pt‐ZnO/CHO preparation and its mechanism for Breast Cancer treatment. a) Pt‐ZnO nanoparticles were first synthesized by hydrothermal method, followed by in‐situ growth of Pt nanoparticles on their surface and further loading of CHO, to finally obtain Pt‐ZnO/CHO. b) Pt‐ZnO/CHO initiates a piezoelectric‐catalyzed cascade reaction under US irradiation to generate O_2_ and ROS as well as to deplete cholesterol, destroying the integrity of lipid rafts and thus blocking the migration of tumor cells, enabling highly efficient piezoelectric‐catalyzed anti‐tumor effects.

For this demand, we developed an engineered piezocatalyst‐cholesterol oxidase (CHO)‐loaded Pt‐ZnO nanoparticles (Pt‐ZnO/CHO) for enhanced piezocatalytic therapy of cancer (**Scheme** **1**a). The narrow bandgap of Pt‐ZnO enables efficient activation under US irradiation, facilitating the separation of electron‐hole pairs (e⁻/h⁺). This property, combined with its notable catalase‐like activity, significantly enhances its efficacy in sonocatalytic therapy.^[^ ^23^ ^]^ Specifically, Pt‐ZnO is capable of substantially generating amounts of ROS via triggering piezoelectric catalytic reactions under US irradiation. Such ROS catalytic production mode with H_2_O and O_2_ as substrates could address the hypoxia limitation of TME. Concurrently, the catalase mimetic activity of Pt‐ZnO imparted by Pt nanoparticles facilitates the decomposition of the intra‐tumoral overexpression of H_2_O_2_ into O_2_,^[^ ^24^ ^]^ which further augments the ROS production. More importantly, CHO‐mediated cholesterol depletion can disrupt the structural integrity of lipid rafts on the cell membrane, effectively inhibiting the formation of lamellipodia and consequently obstructing the pathways for tumor cell migration and invasion. And the elevated intratumoral oxidative stress would further exacerbate this process, achieving a synergistically enhanced piezocatalytic therapy for cancer (Scheme 1b). This strategy by boosting ROS generation and blocking the metastatic pathway of cancer cells providing a new option for inhibiting and treating highly metastatic tumors.

## 2 Results and Discussion

### 2.1 Synthesis and Characterization

**Figure 1:**
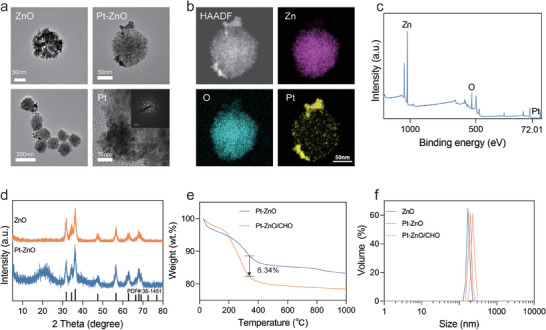
Cauterizations of different materials. a) Representative TEM image of ZnO (top left) and Pt‐ZnO (top right) nanoclusters. b) The elemental mapping of Zn, O, Pt of Pt‐ZnO. c) XPS spectra of Pt‐ZnO nanoclusters. d) XRD patterns of ZnO and Pt‐ZnO powder. e) The thermogravimetry of ZnO and Pt‐ZnO. f) Particle size distributions of ZnO, Pt‐ZnO, and Pt‐ZnO/CHO determined by DLS in PBS.

In this study, mesoporous ZnO nanocrystal clusters were synthesized via high‐temperature decomposition of metal salts at 220 °C. TEM analysis revealed that the synthesized ZnO nanoparticles possess a spherical morphology with an approximate diameter of 150 nm (**Figure** **1**a, top left). Subsequently, Pt nanoparticles were grown in situ on the surface of ZnO nanorods. TEM observations indicated that the morphology and size of the ZnO nanorods remained largely unchanged following the deposition of Pt nanoparticles (Figure 1a, top right). The high‐resolution TEM images reveal that the average size of Pt nanoparticles on the ZnO surface is ≈20 nm (Figure 1a, down right). In the composite Pt‐ZnO, Pt, O, and Zn are uniformly distributed, as revealed by high‐angle annular dark‐field scanning transmission electron microscopy (HAADF‐STEM) (Figure 1b). The X‐ray photoelectron spectroscopy (XPS) survey spectra (Figure 1c; Figure S1, Supporting Information), where Pt, O, and Zn elements were both detected, provide further evidence of the successful construction of Pt‐ZnO. XPS measurements determined the Pt loading quantity to be 5.52%. The energy dispersive spectroscopy (EDS) results corroborated these findings again (Figure S2, Supporting Information). Subsequently, X‐ray diffraction (XRD) analysis was employed to investigate the crystal structure of ZnO before and after Pt deposition. The results indicate that the characteristic diffraction peaks of the synthesized nanorods align well with the standard reference for ZnO (JCPDS No. 36–1451) (Figure 1d). Upon the deposition of Pt nanoparticles, the crystal structure of Pt‐ZnO also corresponds to the orthorhombic phase of ZnO. However, no characteristic diffraction peaks of Pt are discernible in the XRD patterns of Pt‐ZnO. (JCPDS No. 04–0802) (Figure S3, Supporting Information), this phenomenon may be attributed to the minimal quantity of deposited Pt nanoparticles. As Figure S4 (Supporting Information) displayed, the N_2_ adsorption‐desorption isotherms of Pt‐ZnO nanoclusters exhibit a narrow H_1_ type hysteresis loop, indicating the mesoporous structure of Pt‐ZnO.^[^ ^25^ ^]^ The Brunauer‐Emmett‐Teller (BET) surface area of Pt‐ZnO was measured to be ≈103.7 m^2^ g^−1^. Furthermore, the pore diameter was estimated to be ≈3.8 nm using the Barrett–Joyner–Halenda (BJH) method. Due to its unique porous structure and large surface area, Pt‐ZnO is likely to be used as a carriers for efficient loading of CHO. The successful loading of CHO was quantified using thermogravimetric analysis, revealing a content of ≈6.34% (Figure 1e). To enhance the hydrophilicity and improve the dispersion of Pt‐ZnO/CHO in solution, polyethylene glycol (PEG) was employed for surface modification. Dynamic light scattering (DLS) technology was employed to measure the hydrodynamic average particle sizes of ZnO, Pt‐ZnO, and Pt‐ZnO/CHO, which were determined to be 173.2, 192.0, and 232.8 nm, respectively (Figure 1f). These findings are consistent with the results obtained from TEM. Furthermore, the zeta potentials of the ZnO, Pt‐ZnO, and Pt‐ZnO/CHO nanoclusters in aqueous suspension were determined to be −6.0, −13.1, and −16.8 mV, respectively, which significant potential variation further demonstrated the successful loading of CHO (Figure S5, Supporting Information). Altogether, the well‐engineered Pt‐ZnO/CHO nanomedicine has been successfully fabricated.

### 2.2 Piezoelectric Performance and ROS Generation Capacity

**Figure 2:**
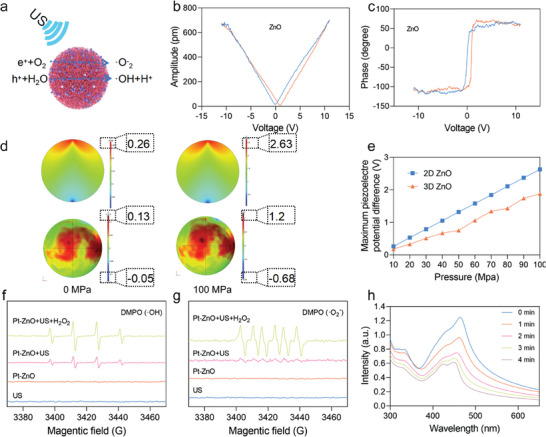
Piezoelectric Performance and ROS Generation Capacity of Pt‐ZnO/CHO. a) Schematic diagram of the ZnO nanoparticles‐mediated piezoelectric effect for the generation of ·OH and ·O_2_^−^. b) Amplitude‐voltage curves and c) phase hysteresis loops of ZnO. d) The calculated distribution of piezoelectric potential and e) the piezoelectric potential difference in 2D ZnO and 3D ZnO under stress ranging from 10 to 100 MPa. f) ESR spectra of DMPO/·OH and g) DMPO/·O_2_^−^ for the Pt‐ZnO nanoclusters with different treatments. h) DPBF as a fluorescent probe to assess the overall ROS generation capacity of Pt‐ZnO.

In order to assess the theoretical suitability of Pt‐ZnO/CHO as a piezoelectric material for tumor treatment, a comprehensive analysis of its piezoelectric properties is required. Firstly, the electronic structures of ZnO were investigated. As shown in **Figure** **2**a, the piezoelectric effect mediated by ZnO nanoparticles facilitated the generation of ·OH and ·O_2_^−^. The piezoelectricity of ZnO was directly detected using piezoresponse force microscopy (PFM). A characteristic butterfly amplitude loop (Figure 2b) and an ≈180° phase switching (Figure 2c) were observed under a bias voltage ranging from ‐10 to 10 V,^[^ ^26^ ^]^ indicating the significant piezoelectric properties of ZnO. Finite element modeling (FEM) simulations were employed to evaluate the strength of the nanoelectric field generated through the US‐mediated piezoelectric effect. Figure 2d and Figure S6 (Supporting Information) displayed the distribution of the piezoelectric potential in both 2D and 3D ZnO structures under applied stresses ranging from 10 to 100 MPa. As illustrated in Figure 2e, the observed minimum and maximum piezoelectric potential differences for the 2D ZnO were 0.26 V (under 0 MPa stress) and 2.63 V (under 100 MPa stress), respectively. In comparison, the 3D ZnO exhibited minimum and maximum piezoelectric potential differences of 0.18 V (under 0 MPa stress) and 1.88 V (under 100 MPa stress), respectively. These potential differences are adequate to induce band tilting in ZnO nanoparticles, thereby rendering the piezocatalytic redox reaction energetically favorable for the generation of ·OH and ·O_2_^−^ species. To investigate the generation of ROS by Pt‐ZnO under US, electron spin resonance (ESR) measurements were initially conducted to directly confirm the production of ROS, such as ·O_2_^−^ and ·OH. As Figure 2f shown, quartet intense characteristic ESR signals with an intensity ratio of 1:2:2:1 were observed when DMPO was employed as the ·OH‐trapping agent, unequivocally indicating the generation of ·OH radicals. Subsequently, the capacity of Pt‐ZnO to generate ·O_2_^−^ under US irradiation was investigated by using ESR measurements. As Figure 2g shown, characteristic ESR peaks with an intensity ratio of 1:1:1:1 were detected when Pt‐ZnO was activated by US (1.0 MHz, 1.0 W cm^−2^, 50% duty cycle), suggesting the formation of ·O_2_^−^ species. Notably, the ESR signal intensity of the Pt‐ZnO + US + H_2_O_2_ group was significantly enhanced compared to that of the Pt‐ZnO + US group, the findings suggest an increased production of ·O_2_^−^ and ·OH radicals. Furthermore, we assessed the overall ROS generation capacity of Pt‐ZnO using 1,3‐diphenylisobenzofuran (DPBF) as a fluorescent probe. The observed decrease in DPBF fluorescence intensity indicated substantial ROS production by Pt‐ZnO upon US irradiation (Figure 2h), thereby confirming the effective generation of ROS. A comparable US treatment conducted without Pt‐ZnO did not result in a significant reduction in DPBF fluorescence intensity (Figure S7a, Supporting Information), thereby affirming the role of Pt‐ZnO in ROS production. Moreover, the Pt‐ZnO + H_2_O_2_ + US group demonstrated markedly weaker fluorescence relative to the Pt‐ZnO + US group, suggesting an enhanced generation of ROS in the presence of H_2_O_2_ (Figure S7b, Supporting Information), it also suggests that Pt nanoparticles imparted the catalase mimetic activity to Pt‐ZnO. The degradation of methylene blue (MB) under US irradiation was investigated as a function of time, to monitor US‐triggered ROS production. As the duration of US (1.0 MHz, 1.0 W cm^−2^, 50% duty cycle) irradiation increased, the characteristic absorption peak of MB at 664 nm markedly decreased, suggesting the generation of ROS by the US‐activated Pt‐ZnO nanoparticles‐mediated piezoelectric effect (Figure S8, Supporting Information). As Figure S9 (Supporting Information) shown, ultrasonic stimulation significantly enhanced CHO release. Furthermore, the potential of Pt‐ZnO to generate O_2_ via piezoelectric catalysis was investigated in greater detail. As Figure S10 (Supporting Information) shown, the interaction between ZnO and H_2_O_2_ yielded an almost negligible production of O_2_ when the Pt doping level was 0%. However, a progressive increase in the Pt doping concentration led to a corresponding enhancement in O_2_ generation. Notably, when the Pt doping concentration reached 5.52%, further increases in Pt content did not result in a significant augmentation of O_2_ production. Consequently, a doping concentration of 5.52% was selected for subsequent analyses.

### 2.3 Cytotoxic Effect of Pt‐ZnO/CHO In Vitro

**Figure 3:**
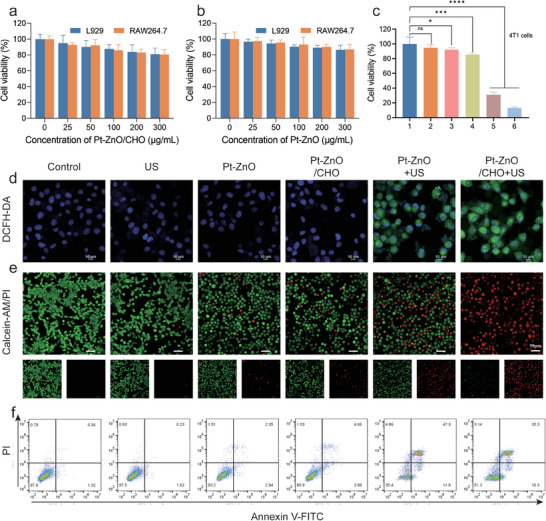
Cytotoxic Effect of Pt‐ZnO/CHO In Vitro. a) CCK‐8 assay results for L929 and RAW264.7 cells following treatment with varying concentrations of Pt‐ZnO/CHO and Pt‐ZnO b) (*n* = 5, mean ± SD). c) CCK‐8 assay results for 4T1 cells subjected to different treatments (*n* = 5, mean ± SD). (1–6 represent: i) Control, ii) US, iii) Pt‐ZnO, iv) Pt‐ZnO/CHO, v) Pt‐ZnO + US, vi) Pt‐ZnO/CHO + US). d) CLSM images depicting total ROS generation detected by DCFH‐DA after various treatments (scale bar: 10 µm). e) CLSM images of 4T1 cells co‐stained with AM/PI following different treatments (scale bar: 50 µm). f) Flow cytometry analysis of apoptosis in 4T1 cells with different treatments. Data are expressed as mean ± SD. Statistical significance was determined by one‐way ANOVA, and ^*^ *p* < 0.05, ^**^ *p* < 0.01, ^***^ *p* < 0.001, ^****^ *p* < 0.0001.

Inspired by the remarkable piezocatalytic performance of Pt‐ZnO/CHO nanocomposites, their therapeutic effects at the cellular level were further investigated. Firstly, the cellular uptake behavior of Pt‐ZnO/CHO was assessed. A Pt‐ZnO/CHO nanocomplex labeled with Cyanine3 (Cy3) was co‐incubated with 4T1 cells for various durations to evaluate their endocytosis potential. Confocal Laser Scanning Microscopy (CLSM) and flow cytometry analyses revealed that the endocytosis capability of the cells for Pt‐ZnO/CHO increased with extended co‐culture time (Figure S11, Supporting Information). The cytotoxicity of Pt‐ZnO/CHO, Pt‐ZnO, and CHO on L929 fibroblast cells and RAW264.7 cells was evaluated in vitro using the standard CCK8 assay. As shown in **Figures** **3**a,b and S12a (Supporting Information) cell viability remained above 80% even at high concentrations of 300 µg mL^−1^ Pt‐ZnO/CHO or Pt‐ZnO or 160 µg mL^−1^ CHO, suggesting minimal cytotoxic effects on healthy cells. Initially, the survival rate of 4T1 cells was assessed under varying US treatment durations, powers, and duty cycles. The US treatment parameters were optimized to 1 MHz frequency, 1 W/cm^2^ power, and a 50% duty cycle (Figure S12b–e, Supporting Information). Additionally, the total ROS generation properties of Pt‐ZnO/CHO were assessed using the ROS probe 2′,7′‐dichlorodihydrofluorescein diacetate (DCFH‐DA). The Pt‐ZnO/CHO + US treatment group exhibited the highest green fluorescence intensity compared to other groups, whereas the Pt‐ZnO + US treatment group showed moderate fluorescence (Figure 3d), the findings indicate that the CHO stimulus exacerbated the production of ROS, aligning with the results obtained from flow cytometric analysis (Figure S13, Supporting Information). In the end, the in vitro anticancer efficacy of Pt‐ZnO/CHO was assessed using the CCK‐8 assay. As Figure 3c shows, 4T1 cells were nearly eradicated under the combined influence of multiple ROS and CHO effects. Furthermore, both acridine orange/propidium iodide (AM/PI) double‐staining (Figure 3e) and flow cytometric analysis (Figure 3f; Figure S14, Supporting Information) corroborated these results. The above results demonstrate that Pt‐ZnO/CHO can produce ROS in an explosive manner through the US‐triggered piezocatalytic effect, thereby inhibiting tumor cells.

### 2.4 Anti‐Metastasis of Pt‐ZnO/CHO In Vitro

**Figure 4:**
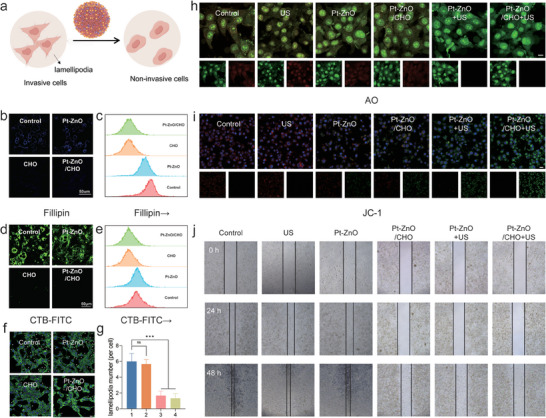
Anti‐metastasis of Pt‐ZnO/CHO In Vitro. a) Schematic illustration of reduced tumor invasiveness by disrupting lipid raft integrity and inhibiting invasive lamellipodia formation. b,c) Cholesterol depletion measured by Filipin III probe (scale bar: 50 µm). d,e) Assessment of lipid raft integrity by CTB‐FITC probe (scale bar: 50 µm). f) Immunofluorescence of F‐actin and changes in lamellipodia in different treatment groups (asterisk indicates lamellipodia, scale bar: 20 µm). g) Statistical results of changes in the number of lamellipodia (per cell) of the 4T1 cells (*n* = 4, mean ± SD). h) Detection of the integrity of the lysosomal membrane by acridine orange (AO) (scale bar: 10 µm). i) Mitochondrial membrane potential (MMP) of 4T1 cells after different treatment stained by 5,5′,6,6′‐tetrachloro‐1,1′,3,3‐tetraethyl‐imidacarbocyanine iodide (JC‐1) probe (scale bar: 20 µm). j) Inhibitory effect of Pt‐ZnO/CHO on cell invasion and migration assessed by wound healing assay. Data are expressed as mean ± SD. Statistical significance was determined by one‐way ANOVA, and ^*^ *p* < 0.05, ^**^ *p* 0.01, ^***^ *p* 0.001.

Metastasis is the primary cause of cancer‐related mortality.^[^ ^12^ ^]^ Consequently, we conducted an in‐depth investigation into the inhibitory effects of Pt‐ZnO/CHO on cellular metastasis. The formation of lamellipodia and the maintenance of lipid raft integrity are essential for tumor cell motility and migration (**Figure** **4**a). Cholesterol, a principal component of lipid rafts, can be efficiently depleted by CHO. This depletion not only disrupts the integrity of lipid rafts but also significantly impedes the proliferation and migration of cancer cells. The Filipin III probe is predominantly employed to detect cholesterol within cellular membranes.^[^ ^27^ ^]^ As Figure 4b,c and Figure S15a (Supporting Information) shown, there was no significant change in the fluorescence signal between the Control group and the Pt‐ZnO group. However, only a weak fluorescence signal was detected in both the CHO group and the Pt‐ZnO/CHO group, suggesting that cholesterol may be consumed by CHO. Lipid rafts, which are highly dynamic, cholesterol‐rich domains within the cell membrane, play a crucial role in various cellular functions, including the regulation of cell membrane movement.^[^ ^18^ ^]^ Subsequently, we investigated whether cholesterol depletion would affect the integrity of lipid rafts. Given that ganglioside GM1 serves as a marker for lipid raft integrity, the cholera toxin B subunit‐FITC probe (CTB‐FITC) was employed to observe its presence in cancer cells.^[^ ^28^ ^]^ As Figure 4d,e and Figure S15b (Supporting Information) displayed, GM1 expression was notably high in cells treated with either the Control or Pt‐ZnO treatment groups. In contrast, a significant reduction in GM1 expression was observed in cells treated with CHO or Pt‐ZnO/CHO, suggesting that cholesterol depletion markedly inhibits the formation of cell membrane lipid rafts. Lamellipodia are expansive, flattened protrusions located at the leading edge of a cell, instrumental in environmental sensing and cellular motility.^[^ ^15^, ^16^ ^]^ Their formation necessitates the assembly of the actin cytoskeleton and the mobilization of the cell membrane.

Consequently, lamellipodia are pivotal in tumor migration and invasion. Given the critical role of lipid rafts in lamellipodia formation, we further examined the potential impact of cholesterol depletion on this process. To this end, we labeled fibronectin (F‐actin) stress fibers using the Actin‐Tracker Green‐488 probe and analyzed the formation of invasive lamellipodia (marked with asterisks) in Figures 4f,g and S16 (Supporting Information).^[^ ^29^, ^30^ ^]^ In the control and Pt‐ZnO treatment group, we observed a pronounced expression of F‐actin stress fibers within the cytoplasm, accompanied by numerous invasive lamellipodia surrounding the cells. Conversely, following treatment with CHO and Pt‐ZnO/CHO, the F‐actin stress fibers appeared less distinct, and membrane protrusions were minimal. This indicates that cholesterol depletion effectively inhibits the formation of invasive lamellipodia. Furthermore, given that cholesterol is a vital component of lysosomal membranes, its depletion also compromises lysosomal membrane integrity, as illustrated in Figure 4h and Figure S15c (Supporting Information). Moreover, the ROS generated by Pt‐ZnO/CHO can induce mitochondrial membrane damage, thereby altering membrane permeability and causing a decline in membrane potential (Figure 4i; Figure S15d, Supporting Information). To further assess the inhibitory effects of Pt‐ZnO/CHO on cell invasion and migration, we performed wound healing and transwell invasion assays (Figure 4j; Figures S15e and S17, Supporting Information). The results indicated a significantly slower wound healing rate and lower cell migration rate in the Pt‐ZnO/CHO treatment group compared to the control group, suggesting a substantial reduction in cell motility and migration following Pt‐ZnO/CHO treatment. The TGF‐β signaling pathway is critically involved in cell migration and EMT.^[^ ^22^ ^]^ We examined the effects of cholesterol depletion on the TGF‐β pathway. As illustrated in Figure S18 (Supporting Information), cholesterol depletion via CHO treatment resulted in impaired activation of TGF‐β receptors, alongside a marked inhibition of Erk and P38 phosphorylation. Additionally, the expression of caveolin‐1, a lipid raft marker,^[^ ^31^ ^]^ was reduced following CHO treatment, indicating diminished lipid raft formation. The observed increase in the epithelial cell marker E‐cadherin suggests a suppression of the epithelial‐to‐mesenchymal transition.^[^ ^32^ ^]^ These findings demonstrate that Pt‐ZnO/CHO possesses notable antimetastatic properties.

### 2.5 In Vivo Antitumor Efficacy, Biodistribution, and Biocompatibility

**Figure 5:**
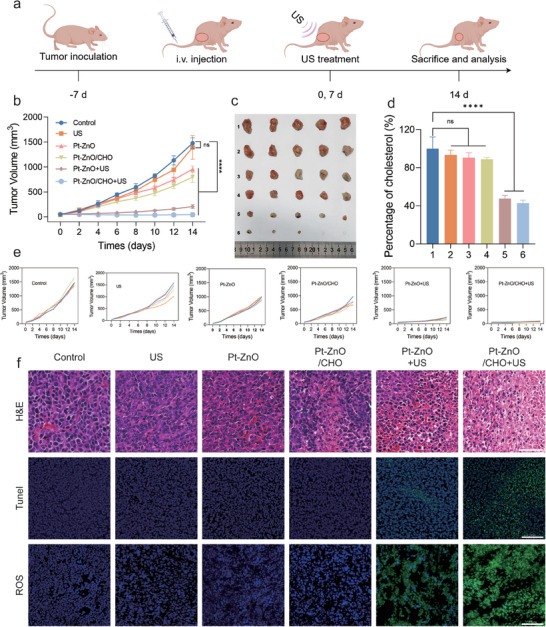
Antitumor performance of Pt‐ZnO/CHO in vivo. a) Schematic representation of the establishment and treatment protocol for the 4T1 tumor‐bearing mouse model. b) Tumor volumes of the mice (*n* = 5, mean ± SD) and c) the corresponding excised tumors of each group on the 14th day in various treatment groups. d) Relative cholesterol content in tumor tissues following various treatments (*n* = 3, mean ± SD, 1–6 represent: i) Control, ii) US, iii) Pt‐ZnO, iv) Pt‐ZnO + US, v) Pt‐ZnO/CHO, vi) Pt‐ZnO/CHO + US). e) Tumor volume progression curves across different experimental groups (*n* = 5, mean ± SD). f) H&E, TUNEL, and ROS staining images of tumor sections. (scale bar: 100 µm). Data are expressed as mean ± SD. Statistical significance was determined by one‐way ANOVA, and ^*^ *p* < 0.05, ^**^ *p* < 0.01, ^***^ *p* < 0.001, ^****^ *p* < 0.0001.

**Figure 6:**
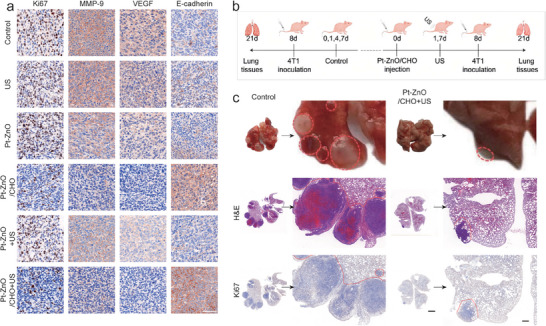
Antimetastasis effects in vivo. a) Histological analysis of tissue sections from primary tumors excised on day 14 was conducted using immunostaining for Ki67, MMP‐9, VEGF, and E‐cadherin (scale bar: 100 µm). b) A schematic representation illustrates the treatment protocol for inhibiting lung metastasis in BALB/c nude mice bearing 4T1 tumors, utilizing US in conjunction with Pt‐ZnO/CHO. c) Representative images of lung tissues from control and Pt‐ZnO/CHO + US treatment groups on day 21 were captured, with metastatic nodules indicated by red circles. (scale bar: 2 mm and 500 µm).

Building on the demonstrated in vitro anticancer and anti‐metastatic efficacy of Pt‐ZnO/CHO, we extended our investigation to evaluate its effects in tumor‐bearing mice (**Figure** **5**a). To assess the real‐time biodistribution and tumor‐targeting capability of Pt‐ZnO/CHO in vivo, the nanoparticles were conjugated with Indocyanine Green (ICG). Fluorescence intensity at the tumor site was subsequently measured at intervals of 1, 2, 4, 8, 12, and 24 h post‐ICG injection (Figure S19a, Supporting Information). The results demonstrated that the fluorescence intensity peaked at 8 h postinjection and maintained a relatively high level even at 24 h (Figure S19b, Supporting Information). Following 24 h of intravenous administration, tumor tissue and major organs (heart, liver, spleen, lung, kidney) were harvested, revealing pronounced fluorescence in the tumor tissue. The liver tissue exhibited moderate fluorescence, whereas other organs did not display significant fluorescence (Figure S19c, Supporting Information). These findings indicate that Pt‐ZnO/CHO possesses a heightened capability for tumor accumulation. Subsequently, we evaluated the in vivo biocompatibility of Pt‐ZnO/CHO. Fifteen healthy BALB/c mice were randomly allocated into three groups (*n* = 5) and received intravenous injections of either saline or different concentrations of Pt‐ZnO/CHO. On the 30th day post‐injection, the mice were euthanized for further analysis. During the 30‐day observation period, no significant differences in body weight were detected among the three groups (Figure S20, Supporting Information). Additionally, histological examination of major organs, including the heart, liver, spleen, lung, and kidney, revealed no notable differences (Figure S21, Supporting Information). The results of blood routine and biochemical analyses did not reveal significant differences among the three groups (Figure S22, Supporting Information). All the above findings suggest that Pt‐ZnO/CHO exhibits good biocompatibility. Subsequently, we assessed the in vivo anticancer efficacy of Pt‐ZnO/CHO in a BALB/c nude mice model to eliminate the confounding effects of immune responses. Five‐week‐old female BALB/c nude mice were utilized for the study. A tumor‐bearing mouse model was established by subcutaneously injecting 2 × 10^6^ 4T1 cells in 100 µL of PBS into the right hind limb area of each mouse. The tumor‐bearing mice were allocated into six groups (*n* = 5): i) Control, ii) US, iii) Pt‐ZnO (15 mg kg^−1^), iv) Pt‐ZnO/CHO (15 mg kg^−1^), v) Pt‐ZnO + US (15 mg kg^−1^), and vi) Pt‐ZnO/CHO + US (15 mg kg^−1^). For the US group, Pt‐ZnO + US group and Pt‐ZnO/CHO + US group, US at a frequency of 1.0 MHz and an intensity of 1.0 W cm^−^^2^ was applied to the tumor site for 3 min, 12 h postinjection. Throughout the 14‐day treatment period, mouse weight and tumor volume were measured bi‐daily. As shown in Figure 5b,c,e, and Figure S23 (Supporting Information), tumor volume exhibited a rapid increase in the Control, US, Pt‐ZnO, and Pt‐ZnO/CHO treatment groups. Conversely, tumor growth was inhibited in the Pt‐ZnO + US and Pt‐ZnO/CHO + US groups, with the most pronounced inhibitory effect observed in the Pt‐ZnO/CHO + US group. This phenomenon was attributed to the piezoelectric material Pt‐ZnO generating ROS under US irradiation, thereby inhibiting tumor growth. Furthermore, upon CHO loading, the material catalyzed the generation of H_2_O_2_ from cholesterol within tumor tissues. This H_2_O_2_ was subsequently converted by Pt into O_2_, leading to a substantial increase in ROS production, which further suppressed tumor growth. To investigate the US‐triggered capability of Pt‐ZnO/CHO in reversing tumor hypoxia, HIF‐1α immunofluorescence staining was performed to assess HIF‐1α expression in tumorous tissue following various treatments. The fluorescence intensity was found to be weakest in the Pt‐ZnO/CHO + US group (Figure S24, Supporting Information). Importantly, no significant weight loss or organ damage was observed in either the control or treatment groups (Figures S25 and S26, Supporting Information), indicating no apparent adverse effects associated with Pt‐ZnO/CHO. To assess the impact of CHO on cholesterol consumption in tumor tissues, we randomly selected three tumor specimens from each group to measure the total cholesterol content. As shown in Figure 5d, the CHO treatment group and Pt‐ZnO/CHO + US treatment group cholesterol levels significantly decreased, indicating that Pt‐ZnO/CHO could effectively inhibit tumor invasion and metastasis. To evaluate the histological anti‐tumor effects of Pt‐ZnO/CHO, one tumor sample from each group was randomly selected for Hematoxylin and Eosin (H&E) staining, Terminal deoxynucleotidyl transferase dUTP nick end labeling (TUNEL) assay, ROS detection, and Ki67 staining. Compared to other groups, the Pt‐ZnO/CHO + US treatment group exhibited the most severe tissue damage, highest ROS production, and lowest level of cell proliferation tendency (Figures 5f and **6**a, first column, Figure S27, Supporting Information).

### 2.6 Anti‐Metastasis In Vivo

Various cytokines, angiogenic cells, immune cells, and fibroblasts within the extracellular matrix (ECM) play a pivotal role in tumor migration and invasion.^[^ ^33^ ^]^ To substantiate the antimetastatic efficacy of Pt‐ZnO/CHO in vivo, we conducted an investigation into the expression levels of metastasis‐associated proteins in primary tumors, specifically matrix metalloproteinase‐9 (MMP‐9), vascular endothelial growth factor (VEGF), and E‐cadherin, utilizing immunohistochemical staining following various treatments (Figure 6a; Figure S28, Supporting Information). MMP‐9, a member of the zinc‐dependent endopeptidase family, plays a pivotal role in the regulation of ECM degradation and is implicated in metastatic angiogenesis.^[^ ^34^ ^]^ VEGF is fundamental for both lymphangiogenesis and angiogenesis, facilitating the provision of O_2_ and nutrients necessary for tumor cell migration and invasion.^[^ ^35^ ^]^ In the Pt‐ZnO/CHO and Pt‐ZnO/CHO + US treatment groups, a marked downregulation of MMP‐9 and VEGF was observed, potentially attributable to CHO's cholesterol consumption, which may subsequently inhibit choriocapillaris formation. E‐cadherin is crucial for preserving the structural integrity of epithelial tissue, and its downregulation is a key factor in the initiation of tumor cell migration and metastasis.^[^ ^32^ ^]^ In our study, we observed a significant upregulation of E‐cadherin in the Pt‐ZnO/CHO + US treatment group. These findings suggest that, under the combined effects of CHO and ROS, Pt‐ZnO/CHO has the potential to inhibit tumor progression and distant metastasis.

To investigate the impact of Pt‐ZnO/CHO on distant metastasis, we assessed its therapeutic efficacy in tumor‐bearing mice using a lung metastasis model. As Figure 6b shown, 4T1 cells (2 × 10^6^ 4T1 cells in 100 µL of PBS) were intravenously injected into mice that were either treated with Pt‐ZnO/CHO in conjunction with US or left untreated to induce lung metastasis. On the 21st day postinjection, all mice were euthanized, and their lung tissues were excised, with metastatic sites indicated by red circles. As Figure 6c shown, mice treated with Pt‐ZnO/CHO in conjunction with US exhibited a markedly reduced number of lung metastatic foci compared to the untreated control group, which presented with numerous metastatic foci. These findings were corroborated by histological examination of lung tissues using H&E and Ki67 staining. Consequently, these results suggest that Pt‐ZnO/CHO, when activated by US irradiation, effectively inhibits primary tumor growth and suppresses distant metastasis.

## 3 Conclusion 

In this study, we present an engineered piezocatalyst consisting of CHO‐loaded Pt‐ZnO nanoparticles, which demonstrates the capability to generate substantial quantities of ROS and inhibit tumor metastasis, thereby enhancing the efficacy of piezocatalytic tumor therapy. In this platform, Pt‐ZnO initiated the conversion of H_2_O and O_2_ into highly toxic ROS via triggering piezocatalytic reactions under US irradiation. At the same time, the released CHO is rapidly depleted excess cholesterol in cancer cells to disrupt their lipid rafts integrity thereby impeding the formation of invasive pseudopodia and effectively inhibiting tumor metastasis. More importantly, Pt‐ZnO produced O_2_ by disproportionating H_2_O_2_ via its inherent CAT‐mimic properties thereby synergistically boosting ROS generation as well as accelerating cholesterol consumption. Therefore, Pt‐ZnO/CHO demonstrated a strong capacity to inhibit tumor growth and metastasis in in vivo antitumor applications. Such a strategy developed in this work, which utilizes piezocatalysts to generate ROS in combination with cholesterol depletion, is expected to address the limitations is expected to address the limitations of current anti‐tumor metastasis therapies.

## 4 Animal Experiments Ethical Approval

All animal experimental protocols were approved by the Ethics Committee of Nanjing Medical University and were in accordance with the policies of the National Ministry of Health (Approved ID: 2310081).

## 5 Statistical Analysis

All in vitro experiments were performed at least three times. All in vivo studies were conducted after randomization into groups. The data were presented as mean ± standard deviation (S.D.) with the indicated sample size in figure legends. One‐way single factorial analysis of variance (ANOVA) was carried out to ascertain the statistical difference in the data. GraphPad Prism 10.1.2 was used for data statistics and statistical significance calculation. *p* < 0.05 was taken as statistically significant (_*_ *p* < 0.05, ^**^ *p* < 0.01, ^***^ *p* < 0.001, and ^****^ *p* < 0.0001).

## Conflict of Interest

The authors declare no conflict of interest.

## Supporting information



Supporting Information

Supplemental Video 1

## Data Availability

The data that support the findings of this study are available from the corresponding author upon reasonable request.
