# Supplementary material for: Engineered Nanocatalyst‐Enabled Cheolesterol Depletion for Enhanced Tumor Piezocatalytic Therapy
Source: Adv Sci (Weinh). 2025 Feb 18;12(14):2500967. doi: 10.1002/advs.202500967 (PMC11984833; doi:10.1002/advs.202500967)
Supplement: Supplementary file 1 — Supporting Information [file ADVS-12-2500967-s002.docx]

Supporting Information

**Chemicals.** Zinc nitrate, manganous nitrate, diethylene glycol (DEG), chloroplatinic acid hydrate (H_2_PtCl_6_), Sodium borohydride (NaBH_4_), Cholesterol Oxidase(CHO), 1,3-Diphenylisobenzofuran (DPBF), and Methylene Blue (MB) were purchased from Aladdin Chemical Reagent Co., Ltd. 2,7-dichlorofluorescein diacetate (DCFH-DA), Cell Counting Kit-8 (CCK-8 kit), Calcein/PI Cell Viability/Cytotoxicity Assay Kit (Calcein/PI), Annexin V-FITC Apoptosis Detection Kit (Annexin V-FITC), acridine orange (AO), Mitochondrial membrane potential assay kit with JC-1, Crystal Violet Staining Solution, and Actin-Tracker Green-488 were purchased from Beyotime Inst. Biotech. Filipin III fluorescent probe and cholera toxin B subunit-FITC (CTB-FITC) probe were purchased from Absin. All chemicals were used as received without purification. 4T1 cells were obtained from Procell.

**Synthesis of ZnO.** ZnO nanoclusters were synthesized via a facile one-step high-temperature decomposition of metal salts at 220 °C. Zn (NO_3_)_2_·6H_2_O (1.8 g) was dispersed in diethylene glycol (DEG, 150 mL). Subsequently, 30 μL of Mn (NO_3_)_2_ solution (50 wt% in H_2_O) was added to the above solution and stirred for 15 min at 60 °C to obtain a homogeneously mixed solution. The solution was heated at 220 °C (2 °C/min) under stirring for 12 h. After centrifugation (10000 rpm/min, 10 min), the collected solid was sequentially washed with distilled water and ethanol. After drying at 60 °C for 6 h in a vacuum oven.

**Synthesis of Pt-ZnO and Pt-ZnO/CHO.** 50mL of ZnO (12 mg/mL) and 93 μL of H_2_PtCl_6_ aqueous solution (20 × 10^−3^ M) were mixed and stirred for 1 h. Then, 250 μL of NaBH_4_ (30 × 10^−3^ M) was added and the mixture was stirred for another 1 h. The products were collected by centrifugation and washed three times with deionized water. In order to further load CHO, the Pt-ZnO was added in deionized water (50 mL). Then, Pt-ZnO was redispersed in water and sonicated for 30 min, and the CHO solution (mass ratio: 1:1) was added dropwise during this process. Pt-ZnO/CHO was collected by centrifugation after stirring 12 h.

**Measurements and characterizations:** The transmission electron microscopy (TEM) and high-resolution transmission electron microscopy (HRTEM) of samples were imaged using a FEI Tecnai G2 S-Twin with a field emission gun operating at 200 kV. X-ray diffraction measurements (XRD) were performed on a D8 Advanced diffractometer (Bruker) using CuKa radiation (λ = 0.154 nm). X-ray photoelectron spectroscopy (XPS) spectra were obtained with thermo scientific K-Alpha using Al Kα (1486.6 eV) as the excitation source.

**Extracellular ROS Detection in Solution.** The general ROS level was detected using DPBF as a probe. Briefly, 3 mL of Pt-ZnO/CHO solution containing DPBF (working concentration: 0.1 mg/mL) was exposed to ultrasound (1.0 MHz, 1.0 W/cm^2^), and recorded the absorption intensity at peak 420 nm by a UV-vis spectrophotometer.

**Assess the sonodynamic performance by using MB.** By using MB to assess the sonodynamic performance of Pt-ZnO/CHO. Briefly, 3 mL of Pt-ZnO/CHO solution containing MB (working concentration: 0.1 mg/mL) was exposed to ultrasound (1.0 MHz, 1.0/cm^2^), and recorded the absorption intensity at peak 420 nm by a UV-vis spectrophotometer.

**Cellular uptake.** The uptake of Pt-ZnO/CHO in the 4T1 cells was detected through

the variation of fluorescence of Cy3 with the help of confocal laser florescence scanning microscope (CLSM) and flow cytometry. Firstly, 4T1 cells were inoculated into 6-well (1 × 10^5^ cells per dish) plates or confocal dishes (1 × 10^5^ cells per dish) for 12 h. And Pt-ZnO/CHO was dispersed in water, and the Cy3 solution at a mass ratio of 1:1 was added dropwise during this process at room temperature. Finally, Cy3 labeled Pt-ZnO/CHO was collected by centrifugation after stirring 12 h under dark condition. Then, 4T1 cells were incubated with Cy3 labeled Pt-ZnO/CHO solution for another 0, 1, 2, 4, and 8 h. After that, 4T1 cells were washed with PBS for three times and detected by CLSM and flow cytometry.

***In vitro* Cytotoxicity.** The cytotoxicity was carried out on RAW264.4 and L929 cells by CCK-8 assay, respectively. RAW264.4 or L929 cells were seeded in a 96-well plate to adhere overnight. Next, 100 μL of Pt-ZnO/CHO solution (0, 20, 50, 100, 200, and 300 μg/mL, n = 5) was added into per well and further incubated for 24 h. Then, 100 μL of CCK-8 solution (1 mg/mL) was added, and the cell viability was determined by a microplate reader.

**Intracellular ROS Assay.** The intracellular ROS level was detected through the CLSM and flow cytometry, DCFH-DA was used as a probe. 4T1 cells were inoculated into 6-well (1 × 10^5^ cells per dish) plates or confocal dishes (1 × 10^5^ cells per dish) for 12 h. Then, 4T1 cells were treated with different groups: i) Control, ii) US (1.0 MHz, 1.0 W/cm^2^, 3 min), iii) Pt-ZnO (100 μg/mL), iv) Pt-ZnO/CHO (100 μg/mL), v) Pt-ZnO + US (100 μg/mL), vi) Pt-ZnO/CHO + US (100 μg/mL), and cultured for 6 h. Then, 4T1 cells were washed three times with PBS. Next, the culture medium was replaced with DCFH-DA (10 μM) and cultured for 20 min. After that, the 4T1 cells were detected by CLSM and flow cytometry.

**Intracellular Antitumor Performance.** 4T1 cells were seeded in 96-well plates (1 × 10^5^ cells per well) and cultured for 12 h. Then, 4T1 cells were treated with different groups: i) Control, ii) US (1.0 MHz, 1.0 W/cm^2^, 3 min), iii) Pt-ZnO (100 μg/mL), iv) Pt-ZnO/CHO (100 μg/mL), v) Pt-ZnO + US (100 μg/mL), vi) Pt-ZnO/CHO + US (100 μg/mL), and cultured for 24 h. 4T1 cells were washed three times with PBS, and the activity of cells was tested by Cell Counting Kit-8 assay kit. For flow cytometry analysis and the CLSM images, 4T1 cells (1 × 10^5^ cells per well) were cultivated into a 6-well dish or confocal dishes for 12 h to adhere. Next, 4T1 cells were treated with the same method, after cultured for 6 h, then, the treated cells were treated with an annexin V-FITC/PI apoptosis detection kit or Calcein/PI Cell Viability/Cytotoxicity Assay Kit. At last, the 4T1 cells were detected by CLSM and flow cytometry.

**Detection of invasive lamellipodia around cells.** We labeled F-actin stress fibres with Actin-Tracker Green-488 and further observed the formation of invasive lamellipodia around cells. 4T1 cells were inoculated into confocal dishes (1 × 10^5^ cells per dish) for 24 h. Then, 4T1 cells were treated with different groups: i) Control, ii) Pt-ZnO (100 μg/mL), iii) CHO (40 μg/mL), and iv) Pt-ZnO/CHO (100 μg/mL). After that, the cells were washed by PBS solution and fixed with 4% paraformaldehyde for 10 min at room temperature. Then, the cells were washed with PBS solution and further permeabilized in 0.5% Triton-100 in PBS for 10 min. Then, the samples were washed with PBS solution and incubated with fluorescent phalloidin staining solution 30 min. Finally, the cells were washed twice with PBS and observed by CLSM.

**Intracellular cholesterol depletion.** The depletion of cholesterol was detected by the Filipin III fluorescent probe, which widely used for cholesterol localization on membranes. 4T1 cells were inoculated into 6-well (1 × 10^5^ cells per dish) plates or confocal dishes (1 × 10^5^ cells per dish) for 24 h. Then, 4T1 cells were treated with different groups: i) Control, ii) Pt-ZnO (100 μg/mL), iii) CHO (40 μg/mL), and iv) Pt-ZnO/CHO (100 μg/mL). After 6 h, the treated 4T1 cells were further incubated with Filipin III for 30 min. Finally, 4T1 cells were washed with PBS for three times and detected by CLSM and flow cytometry.

**Detection of lipid raft integrity.** Ganglioside (GM1) is an important marker of lipid raft integrity, so the CTB-FITC probe was further used to observe the expression of GM1 in cancer cells. 4T1 cells were inoculated into 6-well (1 × 10^5^ cells per dish) plates or confocal dishes (1 × 10^5^ cells per dish) for 12 h. Then, 4T1 cells were treated with different groups: i) Control, ii) Pt-ZnO (100 μg/mL), iii) CHO (40 μg/mL), and iv) Pt-ZnO/CHO (100 μg/mL). The treated 4T1 cells were further incubated with CTB-FITC for 30 min. After that, 4T1 cells were washed with PBS for three times and detected by CLSM and flow cytometry.

**Detection of mitochondrial membrane potential (MMP).** The MMP was detected by mitochondrial membrane potential assay kit with JC-1. 4T1 cells were inoculated

into confocal dishes (1 × 10^5^ cells per dish) for 24 h. Then, 4T1 cells were treated with different groups: i) Control, ii) US (1.0 MHz, 1.0 W/cm^2^, 3 min), iii) Pt-ZnO (100 μg/mL), iv) Pt-ZnO/CHO (100 μg/mL), v) Pt-ZnO + US (100 μg/mL), vi) Pt-ZnO/CHO + US (100 μg/mL). The treated 4T1 cells were further incubated with JC-1 for 20 min. After that, the cells were washed by JC-1 buffer solution, and the fluorescence signal was measured by CLSM.

**Detection of lysosomal disruption.** The lysosomal disruption was detected by acridine orange (AO). 4T1 cells were inoculated into confocal dishes (1 × 10^5^ cells per dish) for 24 h. Then, 4T1 cells were treated with different groups: i) Control, ii) US (1.0 MHz, 1.0 W/cm^2^, 3 min), iii) Pt-ZnO (100 μg/mL), iv) Pt-ZnO/CHO (100 μg/mL), v) Pt-ZnO + US (100 μg/mL), vi) Pt-ZnO/CHO + US (100 μg/mL). The treated 4T1 cells were further incubated with AO (10 μM) for 20 min. After that, the cells were washed by PBS buffer solution, and the fluorescence signal was measured by CLSM.

**Cell migration assay.** Cell migration was assessed by a scratch wound assay. 4T1 cells were inoculated into 6-well plates for 24 h. Then, 4T1 cells were treated with different groups: i) Control, ii) Pt-ZnO (100 μg/mL), iii) CHO (50 μg/mL), and iv) Pt-ZnO/CHO (100 μg/mL). Then a scratch wound was created by using a 200 μL pipette tip. Cell culture medium was removed and cells were washed with PBS and then incubated with serum-free culture medium. After 24 hours, photographs were taken at random, and the percentage of wound closure was further calculated.

**Transwell invasion assay.** Firstly, 4T1 cells were treated with different groups: i) Control, ii) Pt-ZnO (100 μg/mL), iii) CHO (50 μg/mL), and iv) Pt-ZnO/CHO (100 μg/mL), and then were made into cell suspensions with serum-free medium. 4T1 cells were moved to the upper well and the bottom chamber was supplemented with medium containing 20% FBS. After 24 h, the 4T1 cells invading the bottom side of the membrane were fixed and then stained with Crystal Violet Staining Solution. Images were captured by optical microscope.

**Western Blotting.** 4T1 cells in a 6-well plate (1 × 10^6^ per well) were exposed to Pt-ZnO or Pt-ZnO/CHO (100 μg/mL) for 6 h. After washing with PBS, cells were treated for US irradiation (1.0 MHz, 1 W/cm^2^, 50% duty cycle) for 3 min. All of the cells were harvested after another incubation for 2 h. Next, the cells were lysed by RIPA lysis buffer, and the amount of extracted protein content was quantified through BCA Protein Assay Kit. The lysates were then loaded in 8% sodium dodecyl sulfate polyacrylamide (SDS-PAGE) gel, which was further transferred to a poly (vinylidene difluoride) (PVDF) membrane. After blocking the membrane by skim milk (5% w/w) in Tris buffer saline with Tween 20 (TBST) for 2 h, the membrane was thoroughly washed with TBST. Immunoblotting was performed by incubating the protein with anti-E-cadherin, anti-Caveolin-1, anti-TGF-βRI, anti-TGF-βRII, anti-P-p38, anti-P-Erk, and anti-ACTIN at 4 °C overnight. Afterward, the membrane was further treated by antigoat HRP secondary antibody for 2 h and examined via Western-Ready enhanced chemiluminescence (ECL) Substrate Kit.

**In Vivo Anticancer Performance.** Animal experiments were set up in six groups (n = 5) as follows: 1) saline, ii) US, iii) Pt-ZnO, iv) Pt-ZnO/CHO, v) Pt-ZnO + US, vi) Pt-ZnO/CHO + US. For in vivo US treatment, the mice were administrated and treated twice on day 0 and day 7 during 14 days of the treatment period. In detail, mice in group iii, iv, v, and vi were intravenous injected with Pt-ZnO or Pt-ZnO/CHO (dosage: 15 mg/kg) on day 0 and day 7, and US irradiation (1.0 MHz, 1.0 W/cm^2^, 3 min) was imposed at 12h post-injection. The tumor volume and body weight of mice were measured every two days. The tumor volume was calculated by the following formula: Volume=length×width^2^ /2. The mice were euthanized, and the tumors were extracted and weighed after 14 days of treatment. Besides, the tumors and main organs (liver, spleen, lung, heart, and kidney) were collected for histopathological analysis via Ki67, E-cadherin, MMP-9 and VEGF, and TUNEL, H&E and ROS for immunohistochemical staining. Depletion of cholesterol in tumor tissues were detected by Total Cholesterol Content Assay Kit. Lung metastasis was established by intravenously inoculating 4T1 cells into the Pt-ZnO/CHO + US or untreated mice on day 8. On day 21, all the mice were euthanized to collect the lung tissue, which were further processed for histopathological analysis via Ki67 and H&E staining.


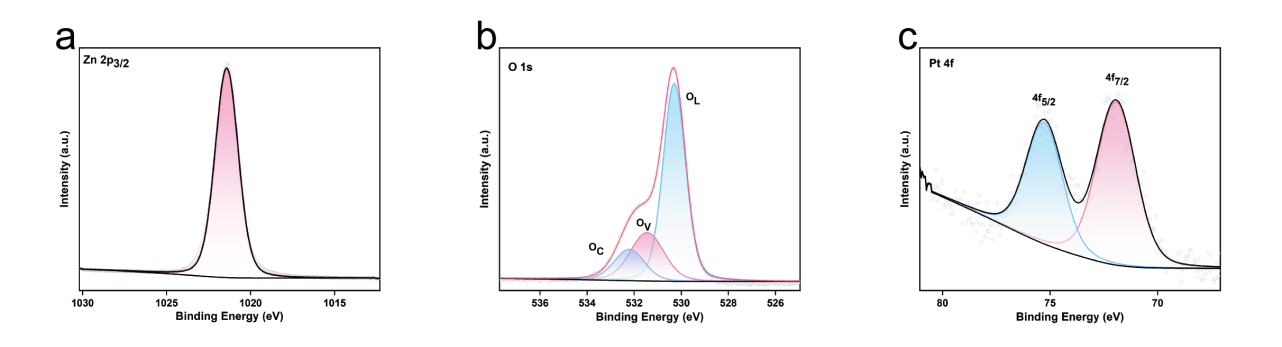


**Figure S1**. High-resolution XPS spectra at Zn 2p (a), O 1s (b) and Pt 4f (c).


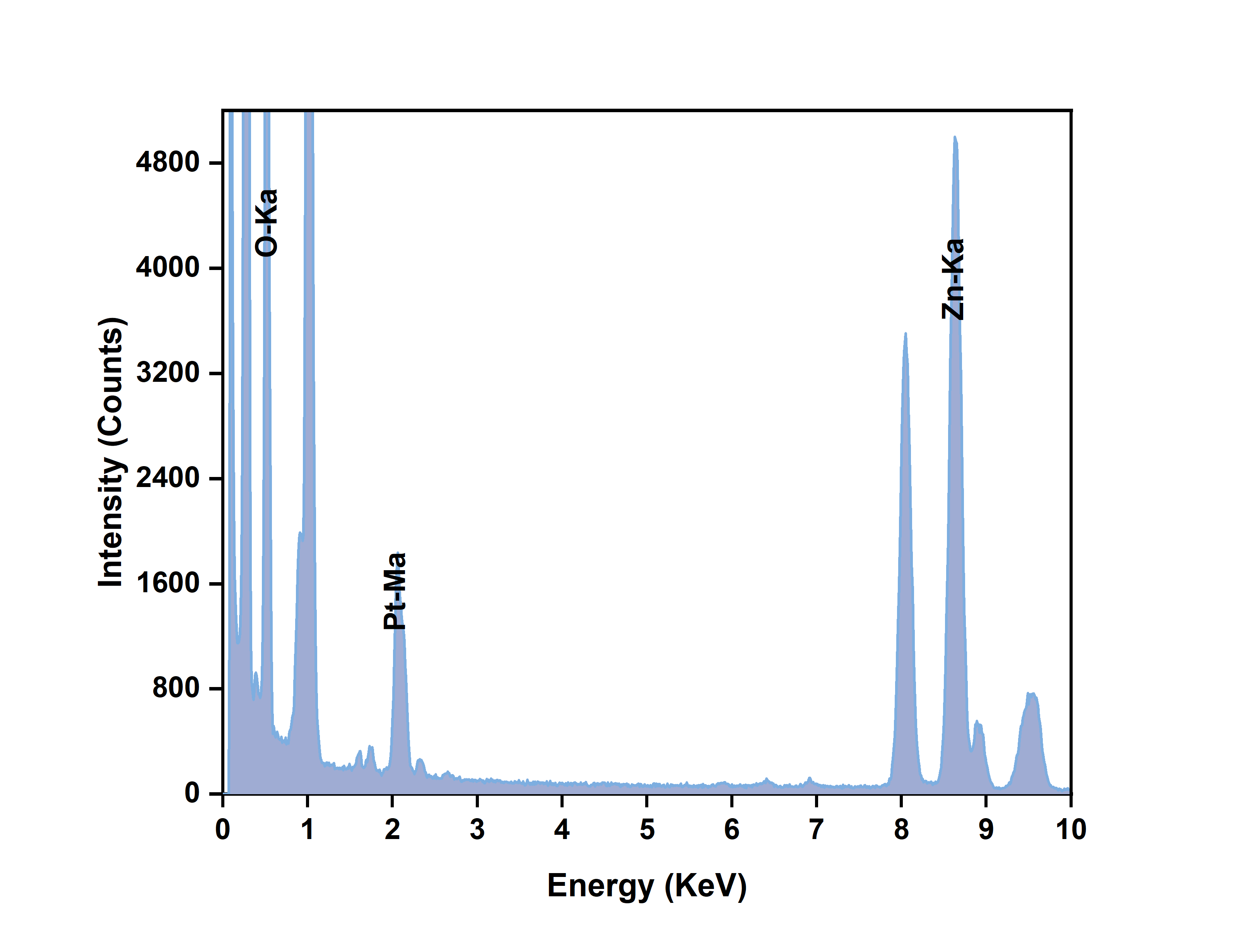


**Figure S2**. Energy dispersive spectroscopy (EDS) profile of Pt-ZnO.


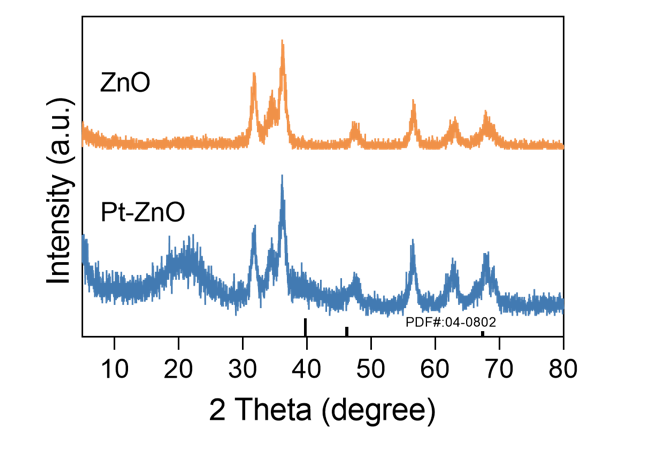


**Figure S3.** XRD patterns of ZnO and Pt-doped ZnO.


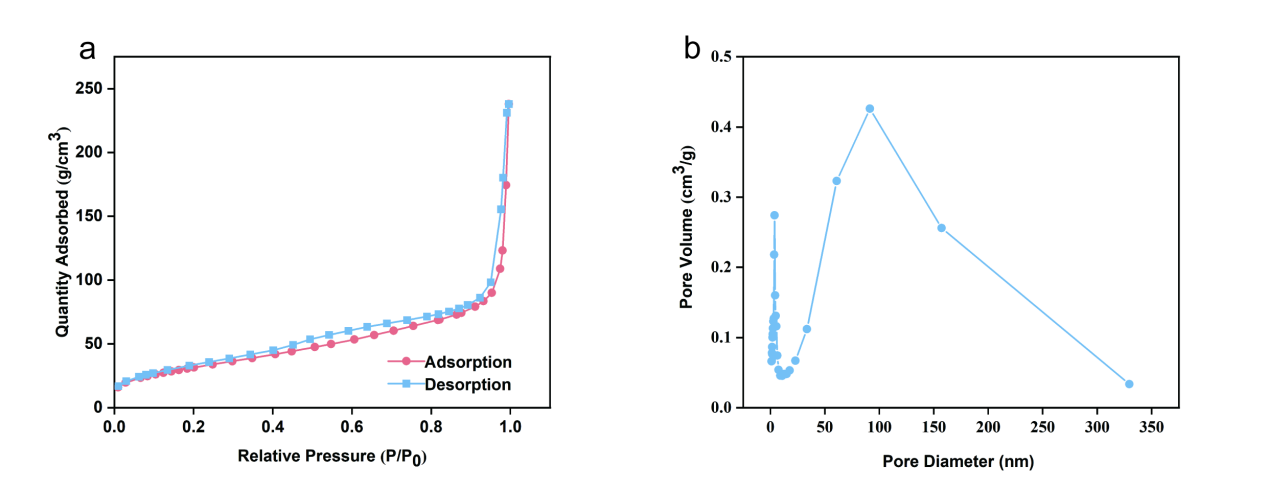


**Figure S4.** (a) N_2_ adsorption/desorption isotherms and (b) pore size distribution of Pt-ZnO.


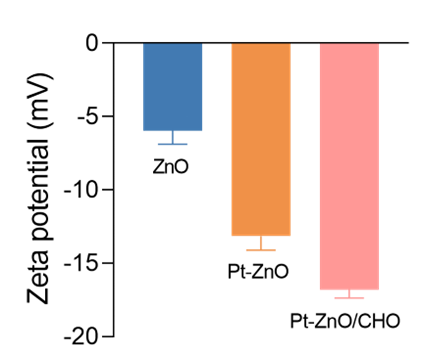


**Figure S5**. Zeta potential of ZnO, Pt-ZnO, Pt-ZnO/CHO (30 µg/mL) in water. Results are represented as mean ± standard deviation (n = 3, mean ± SD).


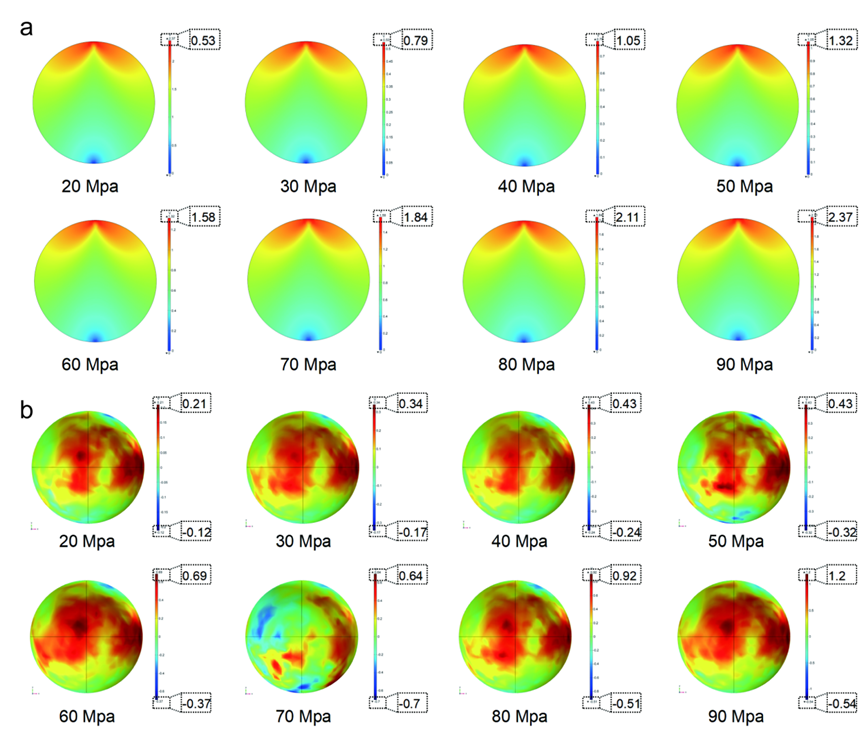


**Figure S6**. The calculated distribution of piezoelectric potential in 2D ZnO (a) and 3D ZnO (b) under stress ranging from 20 MPa to 90 MPa.


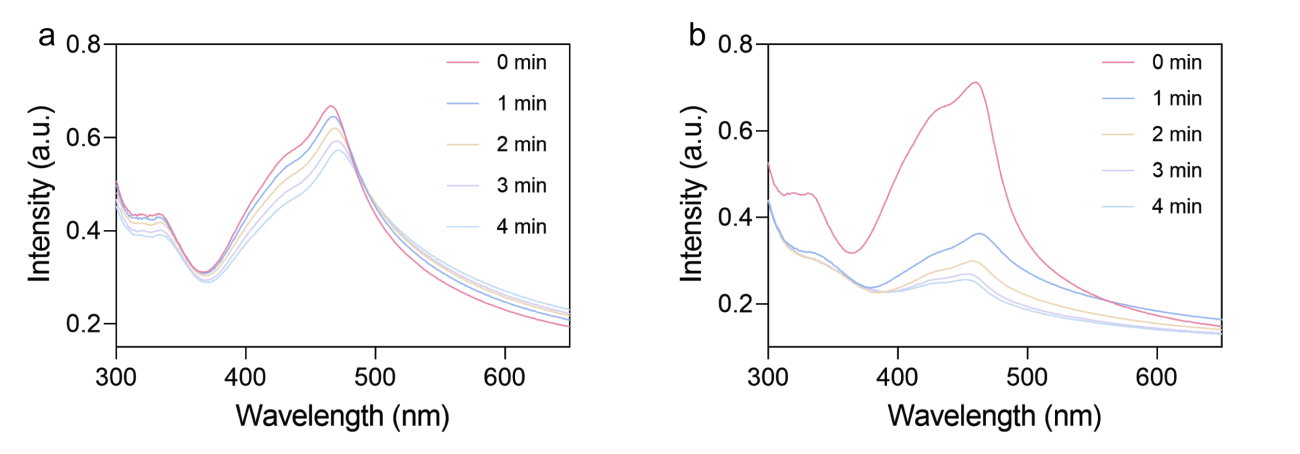


**Figure S7**. UV/vis absorption spectra of DPBF (a) and DPBF + Pt-ZnO + H_2_O_2_ (b) after different US durations.


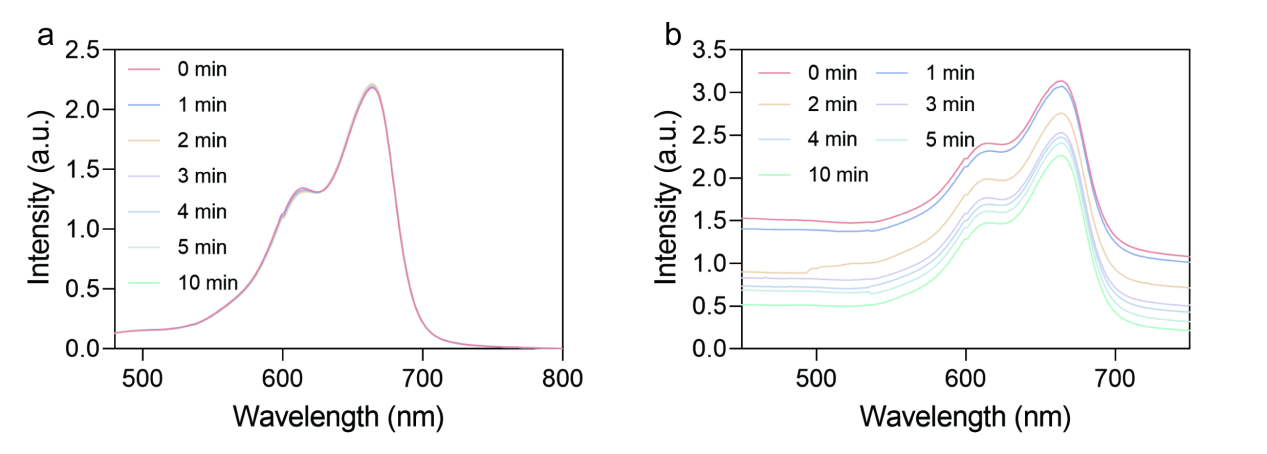


**Figure S8**. UV/vis absorption spectra of MB (a) and MB + Pt-ZnO (b) after different US durations.


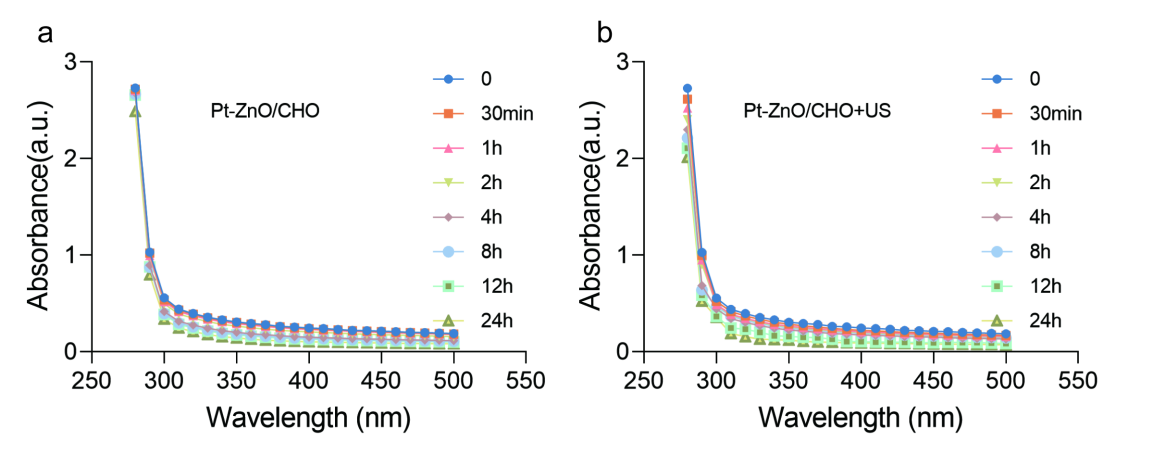


**Figure S9.** Release curves of CHO from the ZnO platform under no US stimulation (a) and with US stimulation (b).


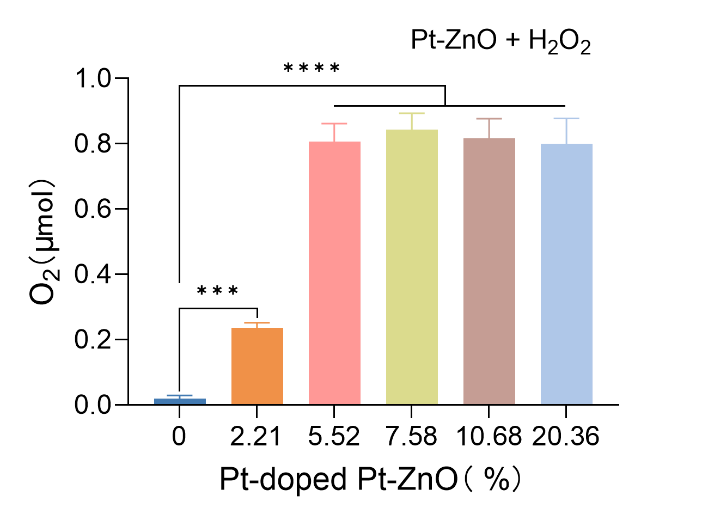


**Figure S10.** Amount of O_2_ produced (μmol) after a five-minute reaction with different amounts of Pt doping at a concentration of 10 mM H_2_O_2_ (n = 3, mean ± SD).


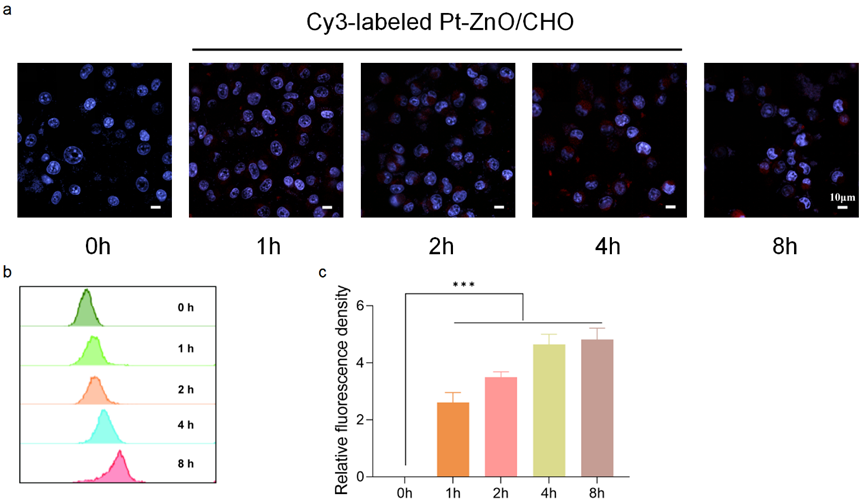


**Figure S11.** CLSM (a) and flow cytometry (b) detected the uptake of Pt-ZnO/CHO in the 4T1 cells at different treatment times. (c) Cy3 fluorescence density in the 4T1 cells at different treatment times (n = 5, mean ± SD).


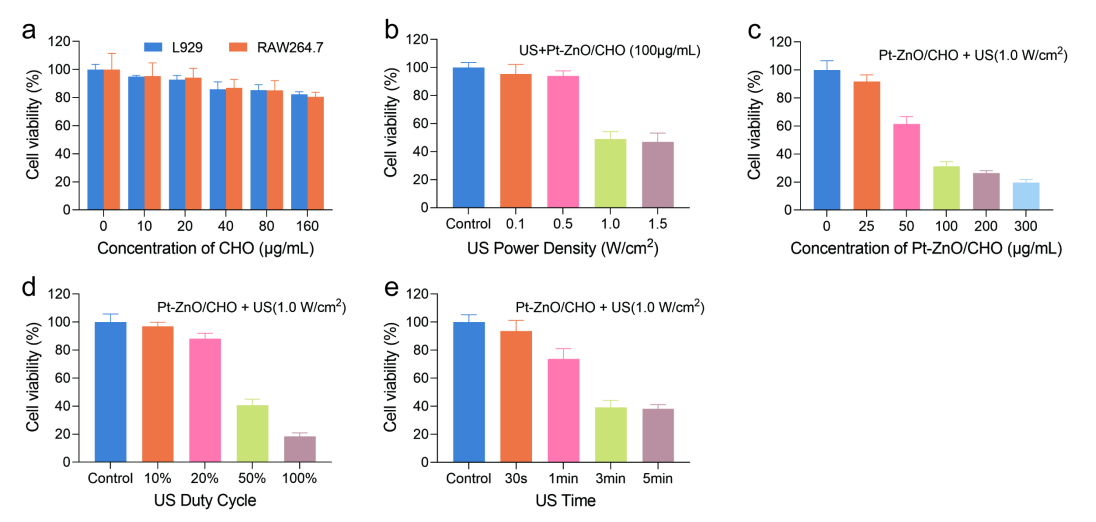


**Figure S12.** (a) CCK-8 assay results for L929 and RAW264.7 cells following treatment with varying concentrations of CHO. The survival rate of 4T1 cells under varying US treatment durations (b), varying Pt-ZnO/CHO concentrations (c), varying US powers (d), and varying US duty cycles (e) (n = 5, mean ± SD).


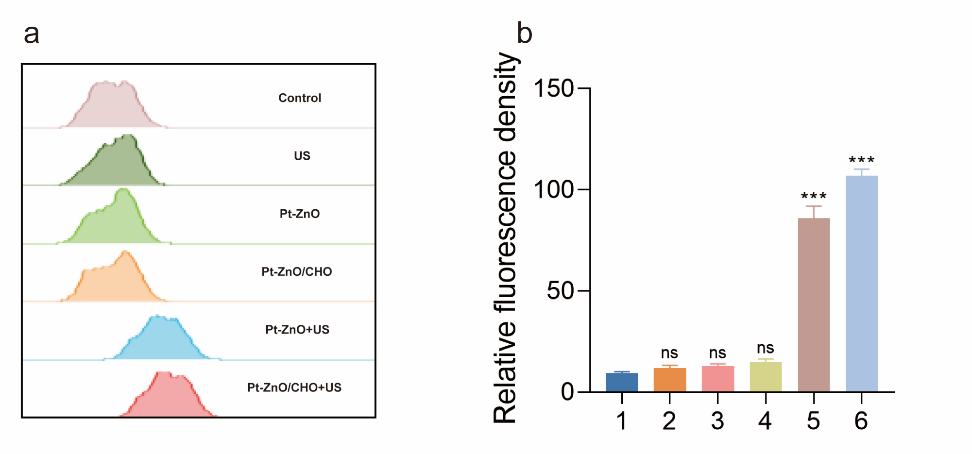


**Figure S13.** (a) Flow cytometry analysis of ROS generation in 4T1 cells with different treatments. (b) DPBF fluorescence density in the 4T1 cells at different treatment groups (n = 3, mean ± SD). Groups were assigned to be (1) Control, (2) US, (3) Pt-ZnO, (4) Pt-ZnO/CHO, (5) Pt-ZnO + US, (6) Pt-ZnO/CHO + US.


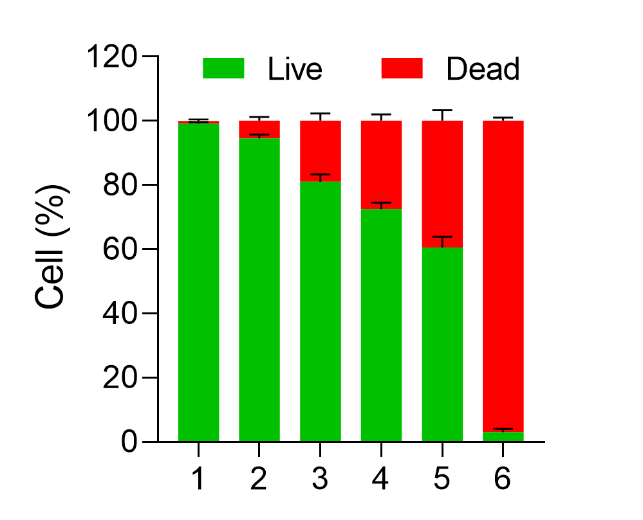


**Figure S14.** Apoptosis of 4T1 cells after different groups (n = 3, mean ± SD). Groups were assigned to be (1) Control, (2) US, (3) Pt-ZnO, (4) Pt-ZnO/CHO, (5) Pt-ZnO + US, (6) Pt-ZnO/CHO + US.


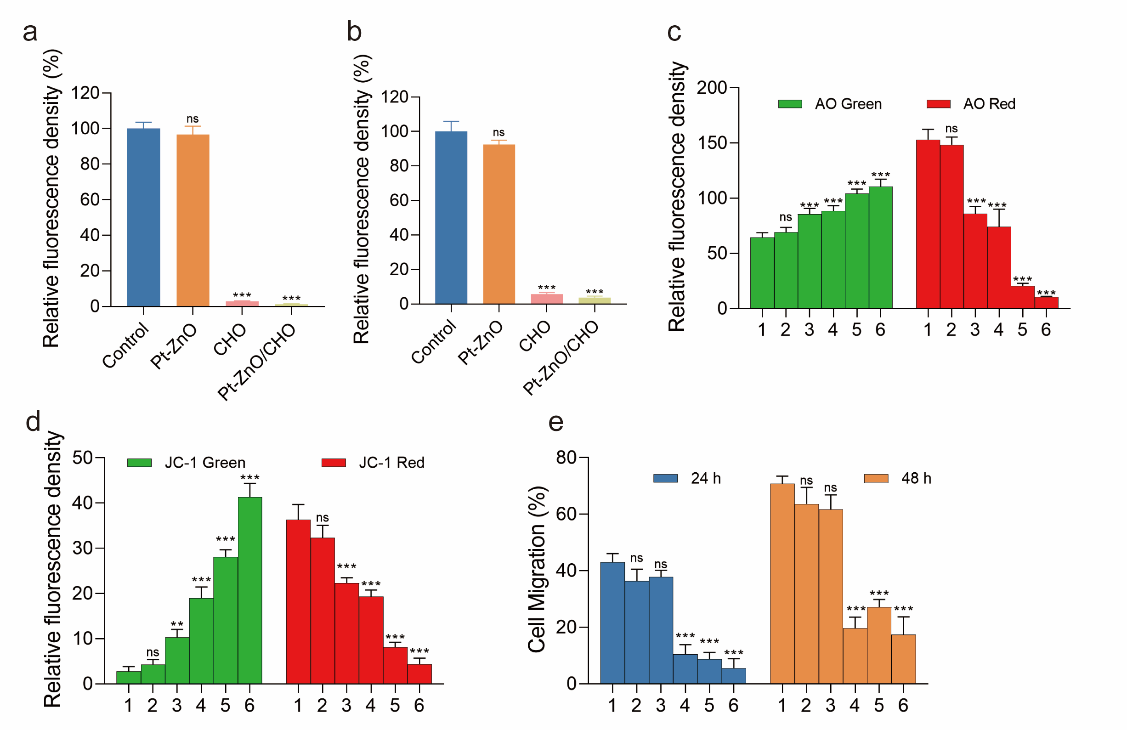


**Figure S15.** (a) CTB-FITC, (b) Filipin III, (c) AO, (d) JC-1 fluorescence density and (e) cell migration in the 4T1 cells at different treatment groups (n = 3, mean ± SD). Groups were assigned to be (1) Control, (2) US, (3) Pt-ZnO, (4) Pt-ZnO/CHO, (5) Pt-ZnO + US, (6) Pt-ZnO/CHO + US.


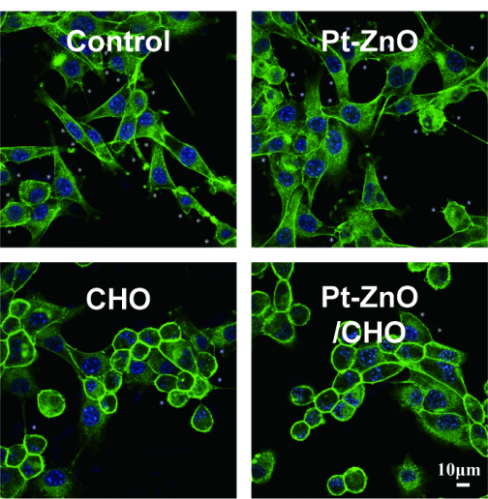


**Figure S16.** Immunofluorescence of F-actin and changes in lamellipodia in different treatment groups (asterisk indicates lamellipodia).


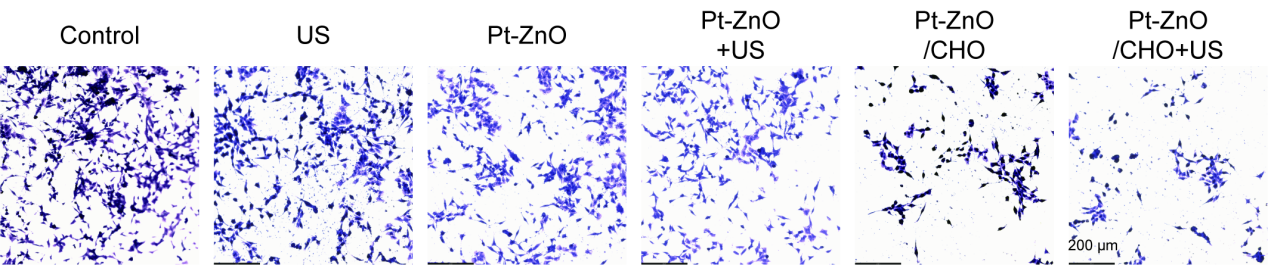


**Figure S17.** Microscope images of 4T1 cells with different treatments, crossing the transwell membrane after incubation for 24 h.


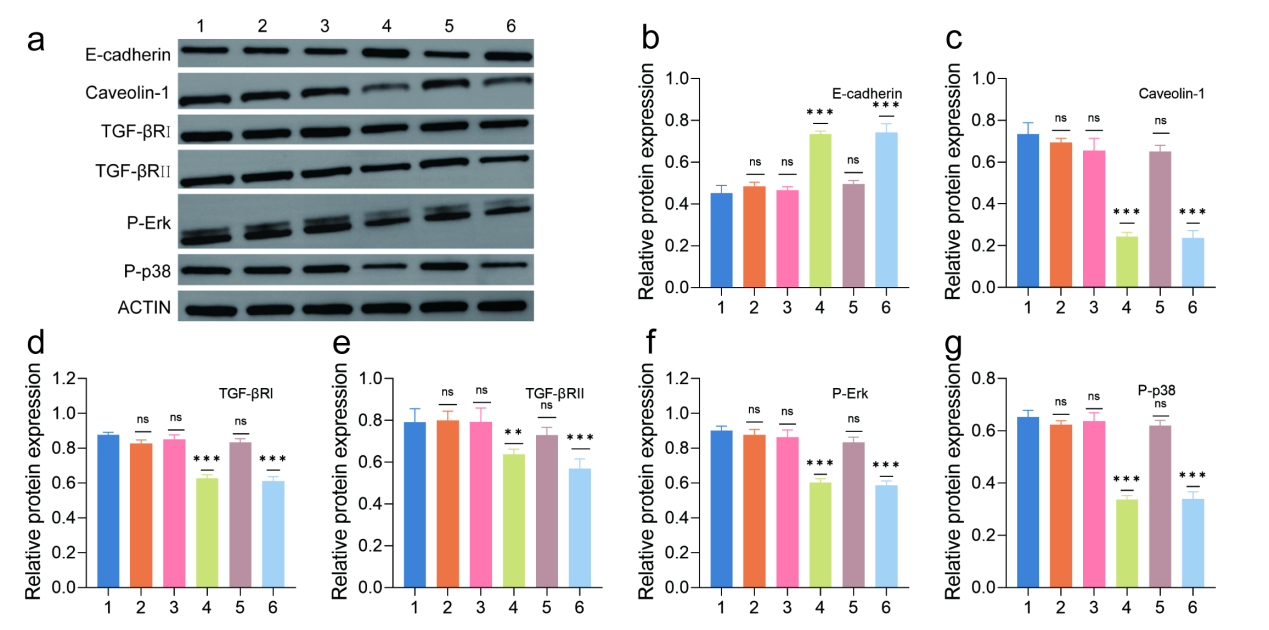


**Figure S18.** (a) Western blotting to indicate the expression levels of E-cadherin, Caveolin, TGF-βRI, TGF-βRII, P-Erk, P-p38 in 4T1 cells from different groups. The relative protein expression (b-g) (n = 3, mean ± SD). Groups were assigned to be (1) Control, (2) US, (3) Pt-ZnO, (4) Pt-ZnO/CHO, (5) Pt-ZnO + US, (6) Pt-ZnO/CHO + US.

**
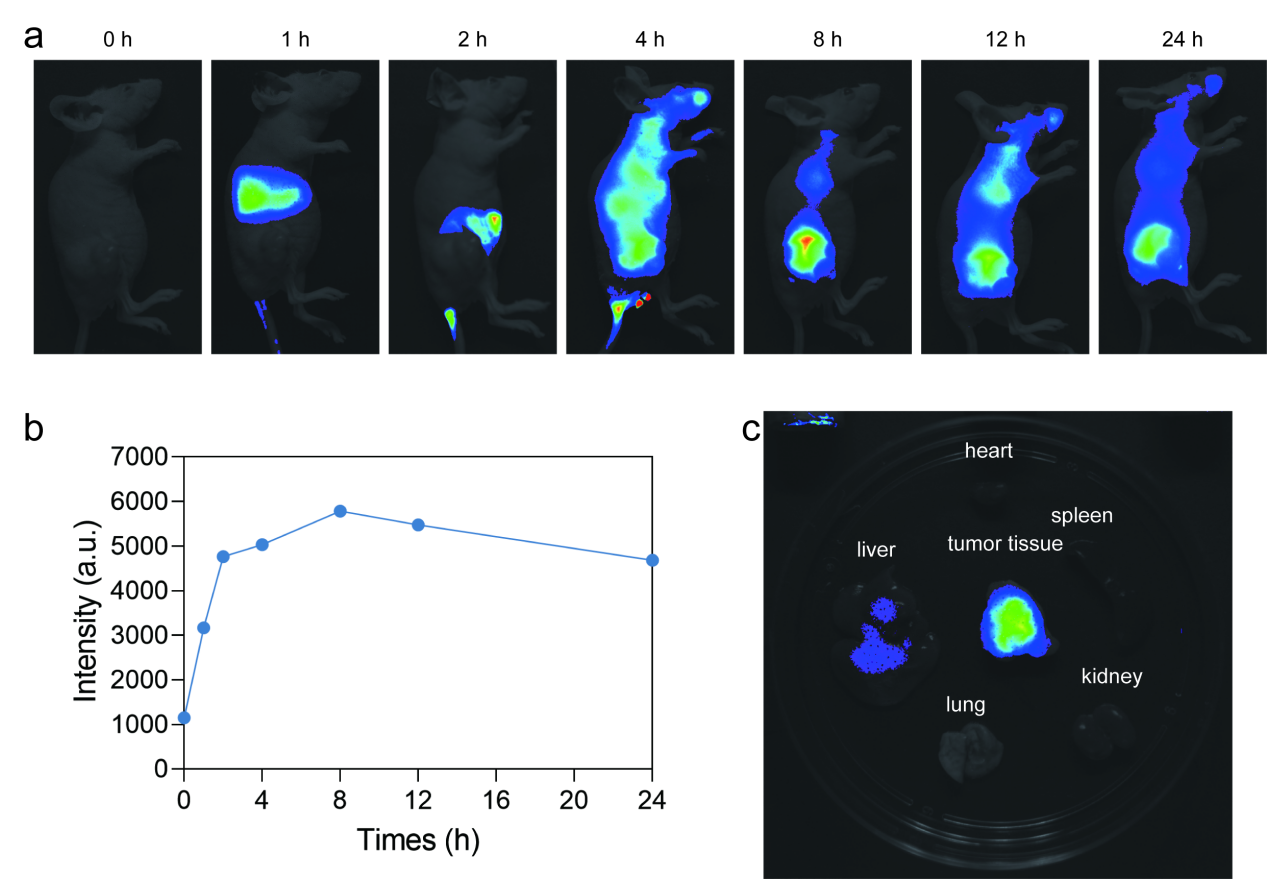
**

**Figure S19.** (a) Images of 4T1-bearing mice after intravenous injection of free ICG-labeled Pt-ZnO/CHO and the change of fluorescence intensity (b) at different times. (c) Ex vivo fluorescence imaging of the tumor and the major organs.


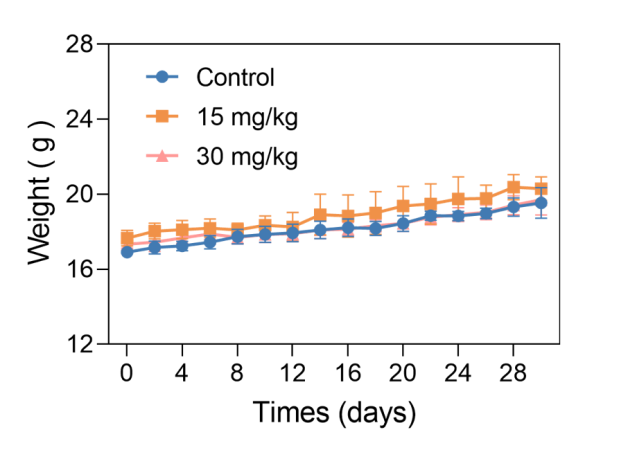


**Figure S20.** Body weights of healthy Balb/c mice after intravenous injection of Pt-ZnO/CHO (dose: 15 mg/kg and 30 mg/kg) at 30 d (n = 5, mean ± SD).


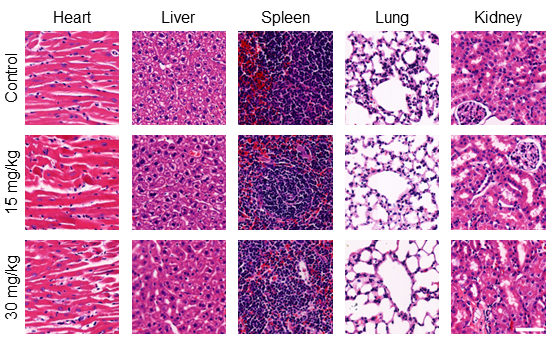


**Figure S21.** H&E staining images of major organs (heart, liver, spleen, lung, and kidney) of healthy Balb/c mice after intravenous injection of Pt-ZnO/CHO (dose: 15 mg/kg and 30 mg/kg) at 30 d (scale bar: 50 μm).


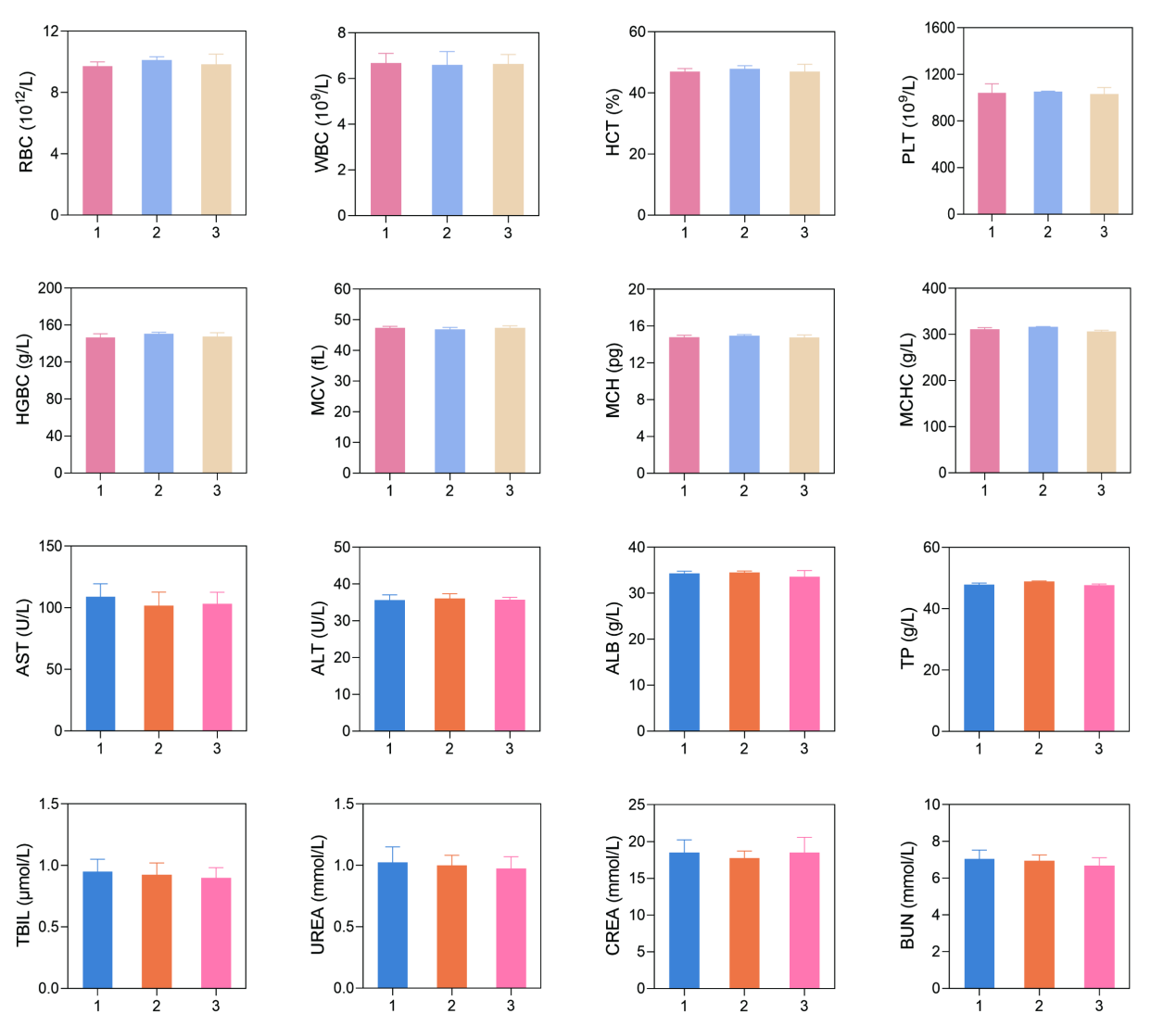


**Figure S22.** Hematological index and biochemical blood analysis of the mice after injection of Pt-ZnO/CHO at 30 d (n = 5, mean±SD, the 1, 2, 3 represent control, 15 mg/kg and 30 mg/kg).

**
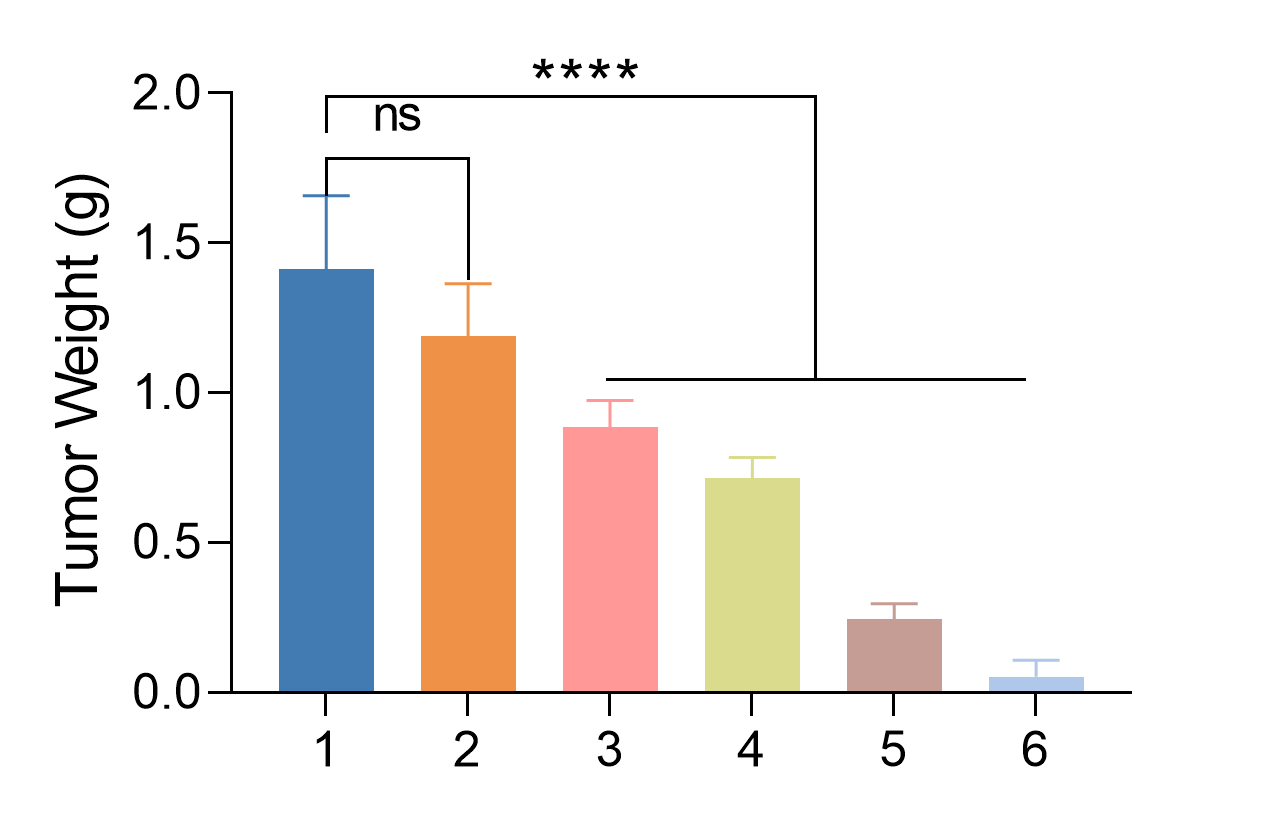
**

**Figure S23.** Tumor weight of the mice following various treatments (n = 5, mean±SD). Groups were assigned to be (1) Control, (2) US, (3) Pt-ZnO, (4) Pt-ZnO/CHO, (5) Pt-ZnO + US, (6) Pt-ZnO/CHO + US.


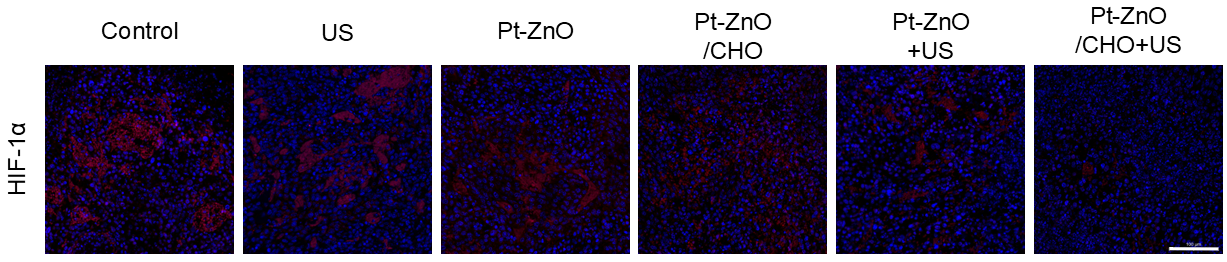
**Figure S24.** Histological analysis of tumor sections through HIF-1α immunofluorescence staining (scale bar: 100 μm).


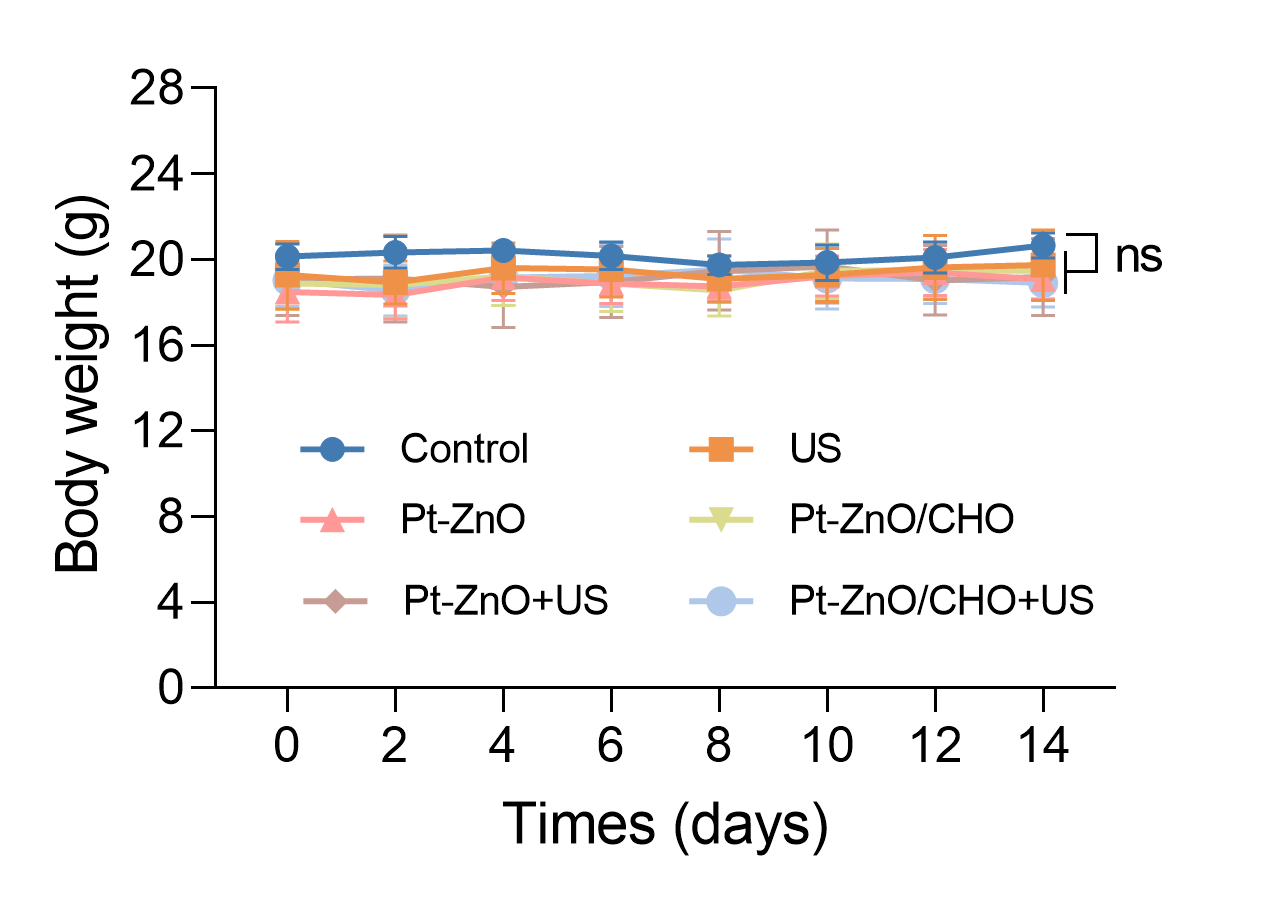


**Figure S25.** Body weights Body weights of mice during different treatments (n = 5, mean ± SD).

**
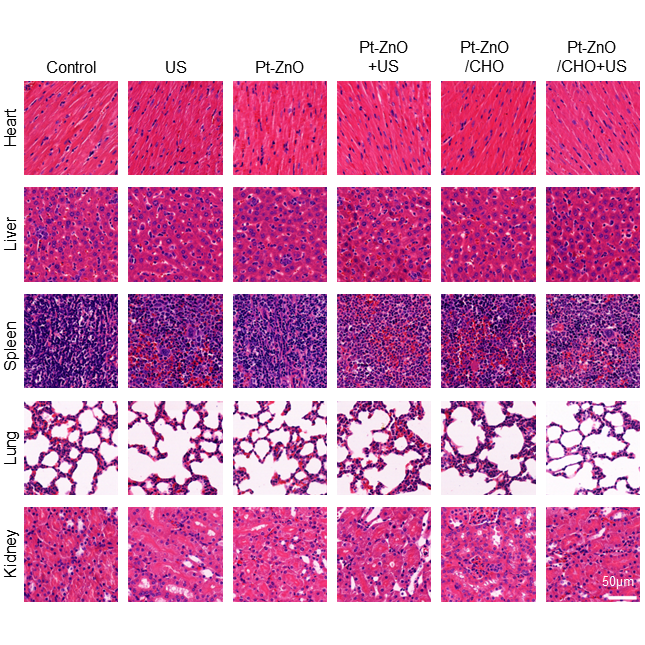
**

**Figure S26.** H&E staining of major organs (heart, liver, kidney, lung, and spleen) after different treatments (scale bar: 50 μm).


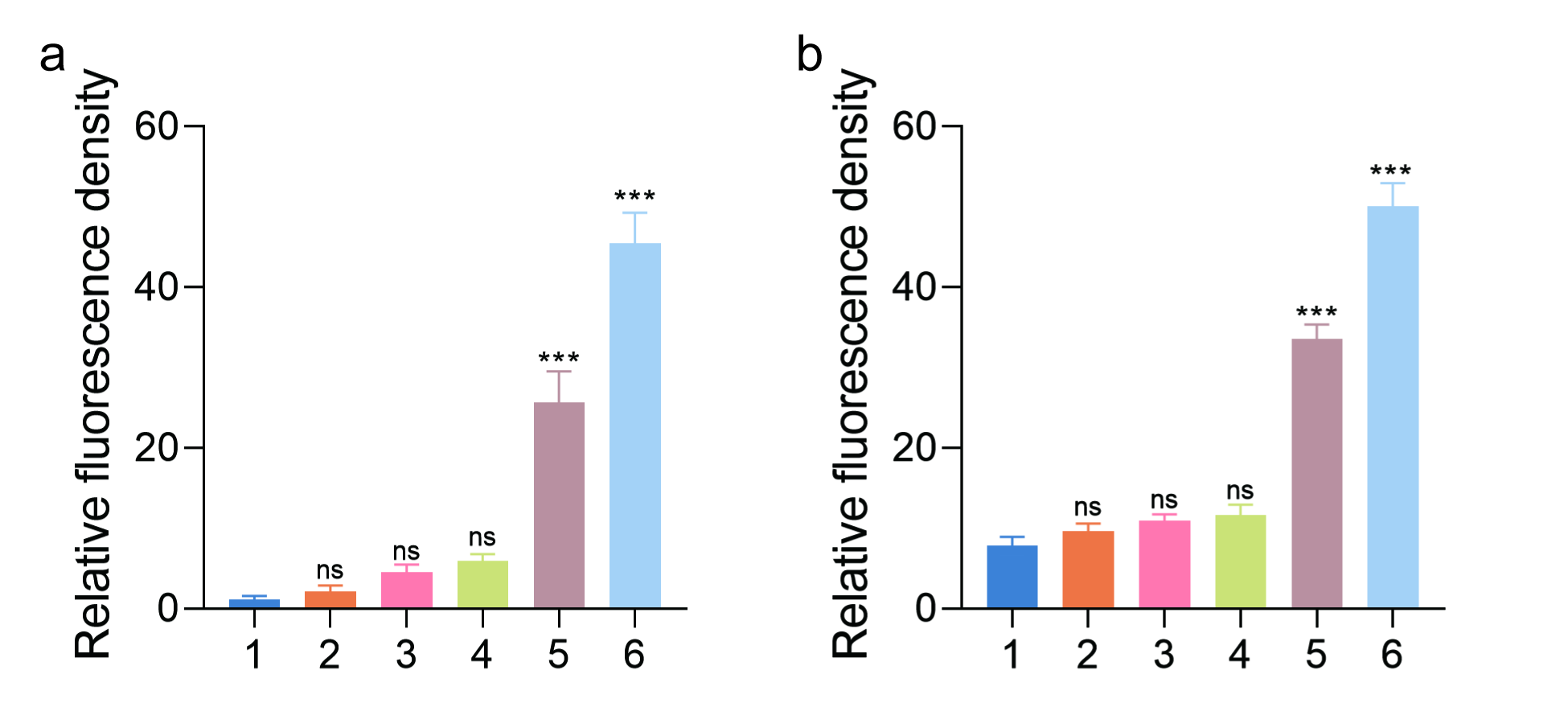


**Figure S27.** (a) Relative fluorescence density of ROS staining and (b) Tunel staining of tumor sections (n = 3, mean ± SD). Groups were assigned to be (1) Control, (2) US, (3) Pt-ZnO, (4) Pt-ZnO/CHO, (5) Pt-ZnO + US, (6) Pt-ZnO/CHO + US.


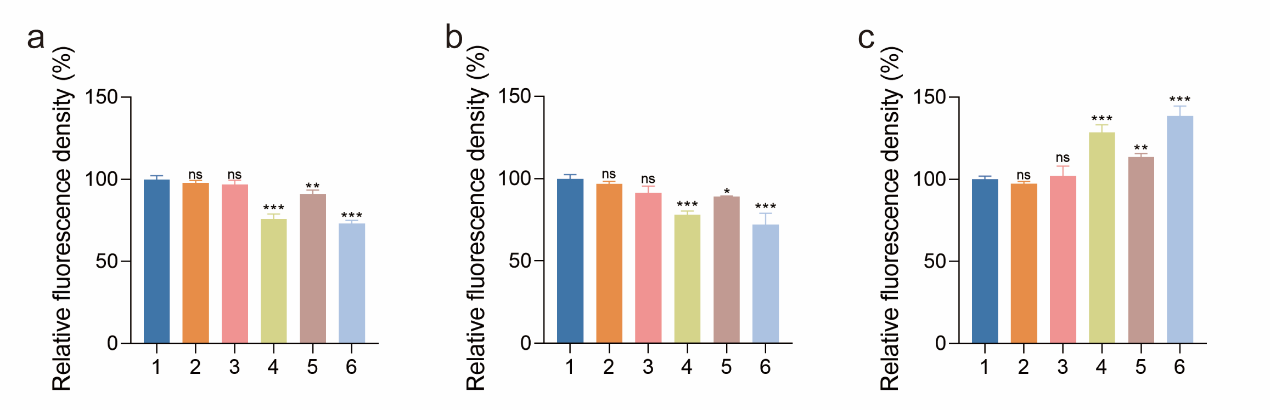


**Figure S28.** (a) Histological analysis of tissue sections from primary tumors excised on day 14 was conducted using immunostaining for MMP-9, (b) VEGF, and (c) E-cadherin (n = 3, mean ± SD). Groups were assigned to be (1) Control, (2) US, (3) Pt-ZnO, (4) Pt-ZnO/CHO, (5) Pt-ZnO + US, (6) Pt-ZnO/CHO + US.
